# Autophagy Underlies the Proteostasis Mechanisms of Artemisinin Resistance in P. falciparum Malaria

**DOI:** 10.1128/mbio.00630-22

**Published:** 2022-04-14

**Authors:** Ananya Ray, Miti Mathur, Deepak Choubey, Krishanpal Karmodiya, Namita Surolia

**Affiliations:** a Molecular Biology and Genetics Unit, Jawaharlal Nehru Centre for Advanced Scientific Research, Bangalore, India; b Life Science Research unit, Persistent Systems Limited, Pune, India; c Department of Biology, Indian Institute of Science Education and Research, Pune, India; NIAID/NIH

**Keywords:** *P. falciparum*, artemisinin, resistance, UPR, PI3P, autophagy, proteostasis, Kelch13, ATG18

## Abstract

Emerging resistance to artemisinin (ART) has become a challenge for reducing worldwide malaria mortality and morbidity. The C580Y mutation in Plasmodium falciparum Kelch13 has been identified as the major determinant for ART resistance in the background of other mutations, which include the T38I mutation in autophagy-related protein *Pf*ATG18. Increased endoplasmic reticulum phosphatidylinositol-3-phosphate (ER-PI3P) vesiculation, unfolded protein response (UPR), and oxidative stress are the proteostasis mechanisms proposed to cause ART resistance. While UPR and PI3P are known to stimulate autophagy in higher organisms to clear misfolded proteins, participation of the parasite autophagy machinery in these mechanisms of ART resistance has not yet been experimentally demonstrated. Our study establishes that ART-induced ER stress leads to increased expression of P. falciparum autophagy proteins through induction of the UPR. Furthermore, the ART-resistant K13^C580Y^ isolate shows higher basal expression levels of autophagy proteins than those of its isogenic counterpart, and this magnifies under starvation conditions. The copresence of *Pf*K13 with *Pf*ATG18 and PI3P on parasite hemoglobin-trafficking vesicles demonstrate interactions between the autophagy and hemoglobin endocytosis pathways proposed to be involved in ART resistance. Analysis of *Pf*K13 mutations in 2,517 field isolates, revealing an impressive >85% coassociation between *Pf*K13 C580Y and *Pf*ATG18 T38I, together with our experimental studies with an ART-resistant P. falciparum strain establishes that parasite autophagy underpins various mechanisms of ART resistance and is a starting point to further explore this pathway for developing antimalarials.

## INTRODUCTION

Despite substantial progress toward malaria elimination in the past 2 decades, it remains a health concern, as 241 million new cases and 627,000 deaths have been registered in 2020 (1). Recovery from Plasmodium falciparum infection relies on treatment with the highly potent artemisinin (ART) and its derivatives. ART rapidly induces a parasiticidal effect that eliminates all intraerythrocytic developmental cycle (IDC) stages of the parasite, yet it is limited by its short half-life of 1 to 3 h (2, 3). This shortcoming is compensated with artemisinin-based combination therapy (ACT), which coformulates fast-acting ART with long-lasting antimalarial drugs (artesunate-pyronaridine, sulfadoxine-pyrimethamine, mefloquine, lumefantrine, piperaquine, or amodiaquine) effective in removing residual parasites (4–6). Regardless of its efficacy, the decreased susceptibility to ART emerging in the southeastern regions of Asia (7, 8), has weakened the effectiveness of the ACT regime, which poses a serious threat for the spread of resistance to other countries where malaria is endemic. Therefore, understanding the molecular mechanisms of ART resistance remains crucial for disease elimination.

Genome-wide association studies and whole-genome sequencing of clinical and laboratory-adapted-ART resistant isolates have identified point mutations in the β-propeller domain of the Kelch13 (*Pf*K13) protein associated with ART resistance (9, 10). The mammalian ortholog of *Pf*K13 functions as a scaffold for ubiquitination and proteasomal degradation of proteins, thereby regulating their cellular levels (11). Also, involvement of mammalian K13 in drug-resistant tumors suggests a similar role of *Pf*K13 in establishing ART resistance in the parasite (12). The major *Pf*K13 variants identified in P. falciparum include C580Y, R539T, Y493H, I543T, and N458Y, with C580Y being prevalent in >50% of parasites across Southeast Asia (10, 13–16). Furthermore, background mutations in genes encoding coroninand the genes listed (*atg18*, *ubp1*, *crt*, *mdr2*, etc.) have also been also reported to regulate the degree of ART resistance (17–20). The early rings (0 to 3 h postinvasion) of *Pf*K13 mutant parasites, when treated with the active ART metabolite dihydroartemisinin (DHA; 700 nM) for 6 h, display >1% survival in ring-stage survival assays (21). ART induces temporary dormancy in rings, and recovery rates are dependent mainly on background genetic mutations (9, 22). The ART activated by heme-derived iron, generated as a result of hemoglobin catabolism, causes widespread cellular damage. Cleavage of the ART endoperoxide bond upon activation releases reactive oxygen species (ROS), which further act promiscuously on protein targets with nucleophilic centers, causing alkylation and eventual parasite death (23–25). Nevertheless, ART-resistant parasites are better equipped to deal with stress imposed upon ART exposure through enhanced protein folding, translation, and vesicular expansion-mediated dissipation of stress-responsive factors attributed to proteostasis (26).

The proposed mechanism for ART resistance unifies the proteostasis pathways in the endoplasmic reticulum (ER) and cytoplasm, which involves phosphatidylinositol-3-phosphate (PI3P) vesicle expansion and the unfolded protein response (UPR) (26–29). As a predicted substrate adaptor for the E3 ligase, *Pf*K13 binds to and ubiquitinates phosphatidylinositol-3-kinase (*Pf*PI3K), facilitating its proteasomal degradation. The *Pf*K13 C580Y mutation prevents *Pf*PI3K ubiquitination and degradation, resulting in increased levels of *Pf*PI3K and its product PI3P (30). Elevation of PI3P in resistance or by transgenically inducing its level increases ER-PI3P vesiculation (27). These *Pf*K13-decorated PI3P vesicles are enriched in proteins related to UPR, folding, quality control, and export. Also, the PI3P-vesicle proteome overlaps with various UPR and oxidative stress responses that are associated with ART-resistant field isolates (26, 27). Additionally, these vesicles contain parasite binding immunoglobulin protein (BiP), which binds to and activates the UPR receptor PKR-like endoplasmic reticulum kinase (PERK) in eukaryotes (31). The activated PERK phosphorylates the elongation factor eIF2α, leading to reduction in the global protein synthesis (32). *Pf*eIF2α phosphorylation-mediated decrease in protein synthesis has also been observed in ART-resistant parasites (33). The amplification of PI3P vesiculation is proposed to be a major determinant of resistance. The model proposed by Suresh et al. integrates the above-described mechanisms to overcome damage from protein alkylation and proteopathy, which lead to parasite death, by proposing expanded ER vesiculation, the major reason for proteostasis (26).

A recent study establishes a link between the *Pf*K13 C580Y mutation and diminished hemoglobin endocytosis by the parasite, proposing another mechanism for ART resistance (34). *Pf*K13 and its associated proteins participate in the parasite hemoglobin endocytosis pathway and thus can regulate hemoglobin uptake. In ART-resistant *Pf*K13 mutant parasites, the concentration of the *Pf*K13 protein is depleted compared to that of ART-sensitive *Pf*K13 wild-type (WT) parasites, and these *Pf*K13-depleted parasites exhibit decreased hemoglobin uptake at the young ring stage (34), leading to dormancy and a consequent delay in progression to the trophozoite stage. Since ART is activated by iron derived from hemoglobin, the diminished availability of hemoglobin confers ART resistance (34).

Each of these pathways, as well as PI3P vesiculation can independently induce autophagy, a cell survival process that involves degradation and recycling of part of the cytoplasm containing protein aggregates and damaged organelles (35). Although PI3P expansion, especially in the ER, stimulates autophagy in other eukaryotes, there is no clear evidence for participation of ER-PI3P vesiculation in inducing autophagy in the proteostasis mechanisms of ART resistance. The malaria parasite has a limited set of conserved autophagy-related (ATG) proteins encoded in its genome (36), such as *Pf*ATG18 (37, 38) and the two ubiquitin-like conjugation systems *Pf*ATG5-*Pf*ATG12 and *Pf*ATG8-PE that are required for autophagosome expansion (39–41). *Pf*ATG8 and *Pf*ATG18 are known to associate with apicoplast biogenesis and are also involved in autophagy-like pathway (37, 38, 40, 41). As a member of the PROPPIN (β-propellers that bind polyphos-phoinositides) family, ATG18 binds to phosphoinositides such as PI3P, which facilitates its localization to the autophagosomes (42, 43). Similar to its yeast counterpart, *Pf*ATG18 also utilizes PI3P for its association with membranes (37). Also, a particular mutation in *Pf*ATG18, T38I, is strongly selected under ART resistance and confers fitness advantage to these parasites (18, 44). Interactions of *Pf*ATG18 with PI3P and the reported *Pf*ATG18 T38I mutation motivated us to carry out studies to understand the role of autophagy in the two proteostasis mechanisms proposed to underlie ART resistance.

This study demonstrates the participation of autophagy in facilitating ART resistance by modulation of various proteostasis functions.

## RESULTS

### ART-activated UPR pathway induces parasite autophagy.

The UPR signaling pathway is activated under ER stress in yeast and other eukaryotes, as well as in P. falciparum (33, 45, 46). UPR acts by initiating its transcriptional and translational stress sensors and adjusting the ER’s capacity, by expansion of its volume, to accommodate protein aggregates and facilitate their refolding (32, 47, 48). To establish UPR activation in the parasite, we incubate synchronized trophozoites (3D7 strain) with the clinically relevant dose of 700 nM DHA for 1 h and 30 min (1.5 h). The results show that ART exposure elicits expansion of the parasite ER space and activation of the UPR translational arm, maintaining ER homeostasis following ART-mediated protein misfolding and damage. The parasite ER extends into the cytoplasm in treated parasites while remaining confined largely to the nuclear periphery in the untreated parasites, as revealed by the confocal and three-dimensional (3D) reconstructed images (see Fig. S1A and B in the supplemental material at https://www.jncasr.ac.in/faculty/surolia/research-highlights/supplemental-material-mbio00630-22). Also, upon incubation of parasites with DHA, a significant upregulation in *Pf*eIF2α phosphorylation is observed (see Fig. S1C). Our results are consistent with previous reports showing DHA-mediated activation of the parasite UPR pathway (33, 46).

**FIG 1:**
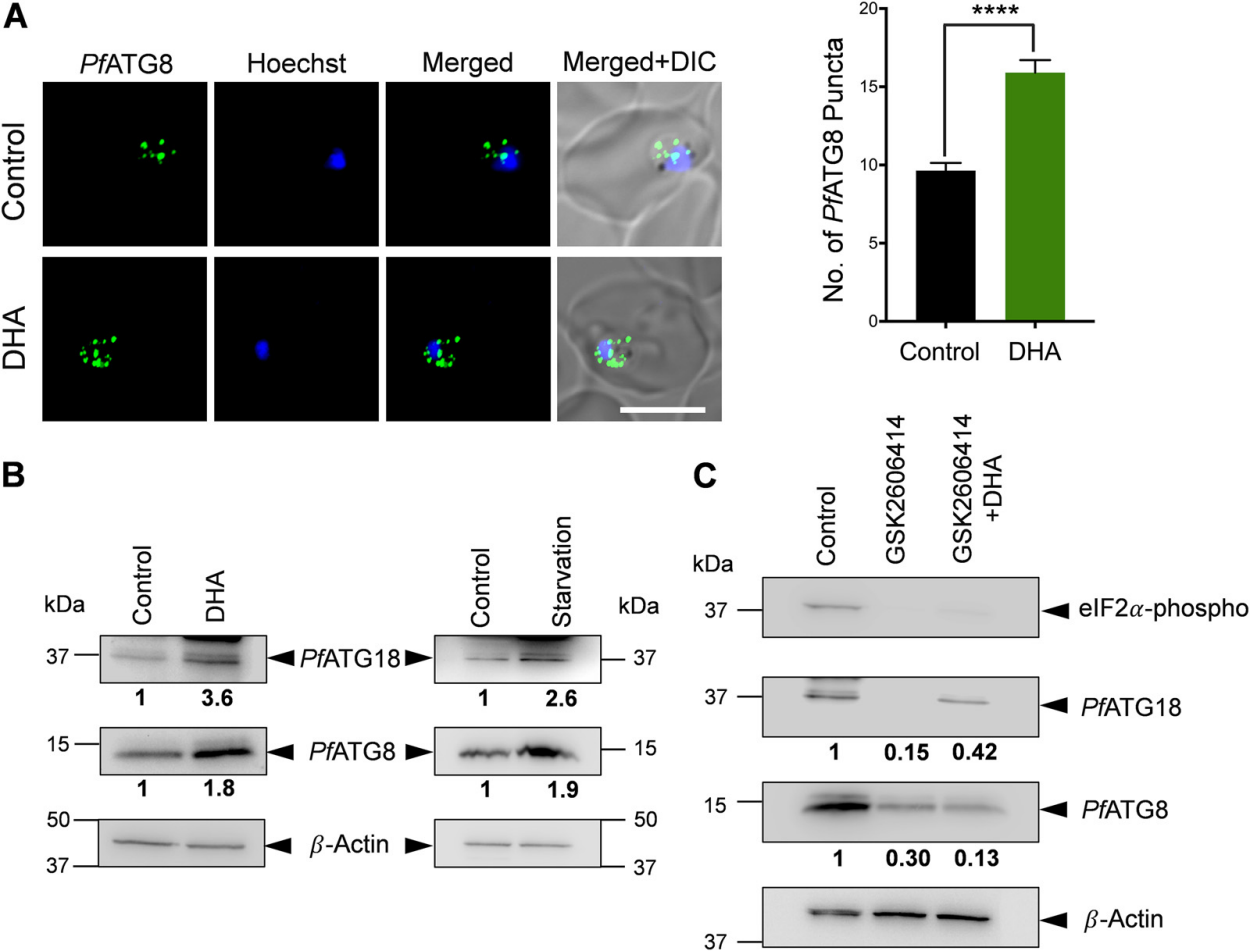
The artemisinin (ART)-activated unfolded protein response (UPR) pathway induces parasite autophagy. (A) Immunofluorescence analysis of P. falciparum 3D7 parasites stained with anti-*Pf*ATG8 antibodies, showing autophagosome-like structures in control and parasites incubated with 700 nM dihydroartemisinin (DHA) for 1.5 h. The nucleus was stained using Hoechst. Graph denoting the numbers of *Pf*ATG8-labeled puncta in the control and in parasites incubated with DHA. *N* > 15 parasites; *n* = 3 independent experiments. Bar, 5 μm. Data points are expressed as mean ± standard error of the mean (SEM). Statistical significance is quantified using the unpaired Student’s *t* test, ****, *P* < 0.0001. (B) P. falciparum 3D7 parasites were incubated with either 700 nM DHA or starvation medium for 1.5 h and harvested. Parasite lysates were subjected to immunoblot analysis, and blots were probed with *Pf*ATG8 and *Pf*ATG18 antibodies. β-Actin was used as the loading control. *n* = 3 independent experiments. (C) P. falciparum 3D7 parasites preincubated with 30 μM GSK2606414 for 1 h were treated with 700 nM DHA for 1.5 h and then harvested. Parasite lysates were subjected to immunoblot analysis, and blots were probed with *Pf*ATG8, *Pf*ATG18, and phosphorylated-eIF2α antibodies. β-Actin was used as the loading control. *n* = 3 independent experiments. Fold difference, normalized with respect to the control, is shown below each blot.

Autophagy is known to counterbalance UPR-mediated ER stress in yeast and higher organisms through sequestration and subsequent degradation of excess ER-containing toxic protein aggregates (47, 49, 50). Our recent studies demonstrated that *Pf*Sec62 is an ER-resident autophagy receptor (51), implicating recovER-phagy as needed to reestablish ER homeostasis upon resolution of ER stress in the parasite. Since there has been no experimental demonstration showing involvement of parasite autophagy upon induction of ER stress, we investigated this topic by incubating young trophozoites with DHA (700 nM, 1.5 h) and analyzed for the presence of autophagosome-like structures. Immunofluorescence analysis for quantifying the number of *Pf*ATG8 (the autophagosome marker)-labeled puncta reveals the number to be significantly higher upon DHA treatment compared to that in the control (Fig. 1A). To investigate whether an enhanced number of autophagosome-like structures correlates with increase in levels of autophagy proteins, the expression levels of *Pf*ATG8 and *Pf*ATG18 were analyzed. As our earlier studies (38, 40) demonstrated increased levels of parasite autophagy proteins upon starvation, parasites were simultaneously incubated with starvation medium as a control. Parasites incubated with DHA display upregulation in the expression levels of *Pf*ATG8 by ∼1.8-fold and *Pf*ATG18 by ∼3.5-fold (Fig. 1B; see also Fig. S2 in the supplemental material at https://www.jncasr.ac.in/faculty/surolia/research-highlights/supplemental-material-mbio00630-22) with respect to the control (untreated), as determined by immunoblot analysis. A similar trend in autophagy modulation is observed under starvation conditions, (Fig. 1B; see also Fig. S2), indicating stress-mediated autophagy induction.

To confirm that the observed increase in expression levels of autophagy proteins upon DHA exposure is directly mediated through UPR, young trophozoites were treated with the mammalian PERK inhibitor GSK2606414, which specifically blocks UPR/PERK activation (52). Expression levels of autophagy proteins were monitored in parasites treated with 30 μM GSK2606414 for 2 h and 30 min (2.5 h). The inhibitor blocks the basal and DHA-induced phosphorylation of *Pf*eIf2α and significantly reduces the expression levels of *Pf*ATG8 and *Pf*ATG18 (Fig. 1C). As autophagy activation is considerably impaired upon disruption of the parasite UPR machinery, it signifies a role in regulating ER stress-induced UPR. These results thus further establish parasite autophagy as an ER stress response pathway in P. falciparum that is triggered upon UPR activation.

### Basal expression of autophagy proteins is higher in ART-resistant isolates.

**FIG 2:**
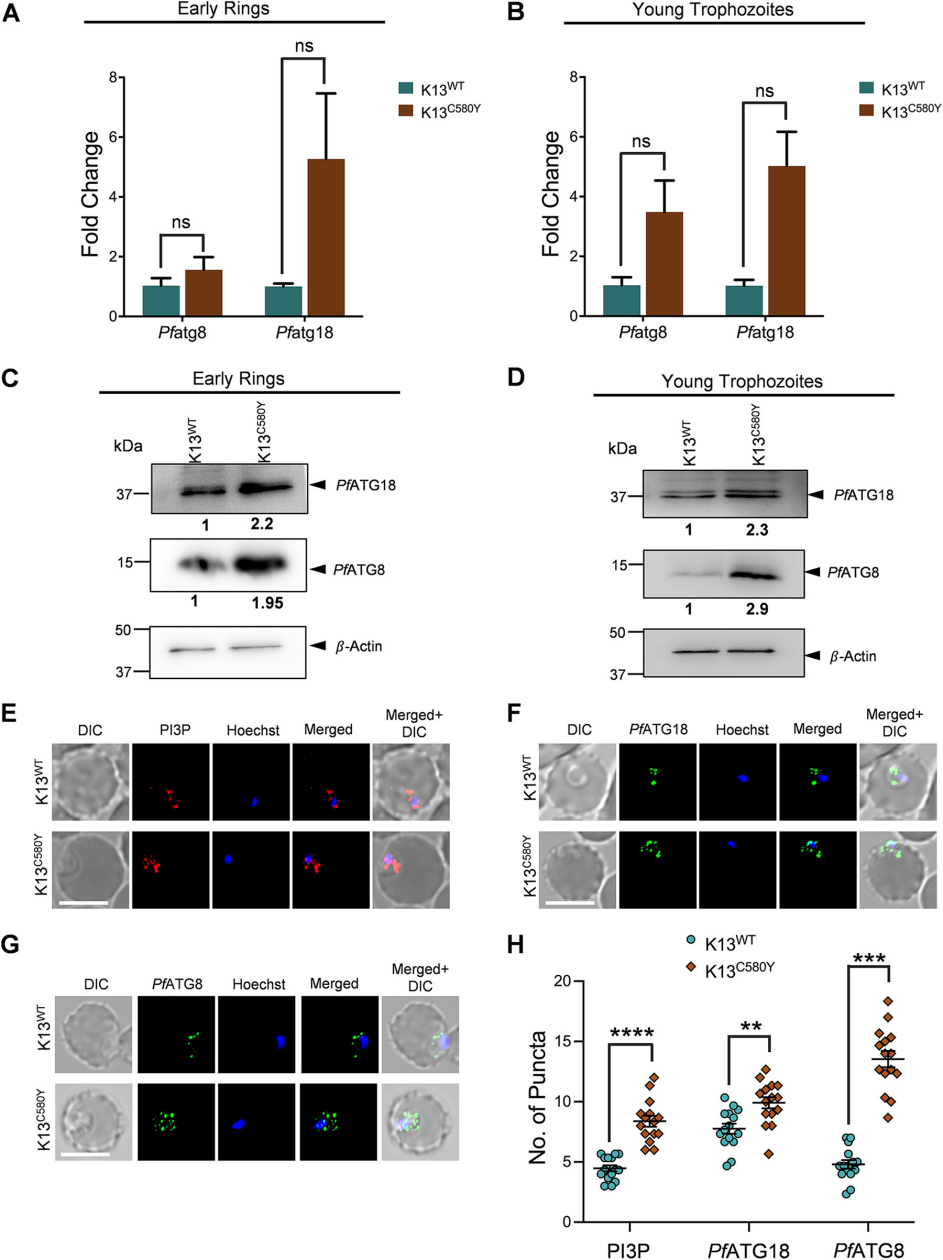
Basal expression of autophagy proteins is higher in ART-resistant isolate. (A and B) Graphs denoting fold changes of *Pf*ATG8 and *Pf*ATG18 gene expression levels in ART-resistant K13^C580Y^ (0 to 3 hours postinfection [hpi]) early rings (A) and young trophozoites (B) compared to those in its isogenic isolate K13^WT^, determined using quantitative real-time PCR. β-Actin was used as the reference gene. Relative gene expression was determined using the comparative threshold cycle (2^−ΔΔC^*^T^*) method. *n* = 2 independent experiments. (C and D) Expression levels of proteins (*Pf*ATG8 and *Pf*ATG18) in K13^WT^ and K13^C580Y^ early rings (C) and young trophozoites (D). Parasite lysates were subjected to immunoblot analysis, and blots were probed with *Pf*ATG8 and *Pf*ATG18 antibodies. β-Actin was used as the loading control. *n* = 3 independent experiments. Fold difference, normalized with respect to the control, is shown below each blot. (E, F, and G) Immunofluorescence analysis of K13^WT^ and K13^C580Y^ parasites stained with anti-PI3P, anti-*Pf*ATG18, and anti-*Pf*ATG8 antibodies showing number of PI3P-labeled puncta (E), *Pf*ATG18-labeled puncta (F), and *Pf*ATG8-labeled puncta (G). The nucleus was stained using Hoechst stain. Bar, 5 μm. (H) Scatterplot showing numbers of PI3P-, *Pf*ATG18-, and *Pf*ATG8-labeled puncta in K13^WT^ and K13^C580Y^ parasites. *N* = 15 parasites, *n* = 3 independent experiments. The data points are expressed as mean ± SEM. Statistical significance is quantified using the unpaired Student’s *t* test; ****, *P* < 0.0001; ***, *P* < 0.0005; **, *P* < 0.005; ns, nonsignificant.

ART-resistant field isolates are known to exhibit increased mRNA levels of genes involved in proteostasis pathways, even at baseline (28). Whether a similar increase in mRNA levels of the parasite autophagy pathway genes is reflected in ART-resistant isolates was investigated. Basal transcription and translational profiles of *Pf*ATG8 and *Pf*ATG18 at the early ring (0 to 3 hours postinfection [hpi]) and young trophozoite stages were compared between two parasite strains, which are matched isogenically but differ only at the *Pf*K13 locus. These are the ART-resistant IPC 3445 strain, a field isolate from Pailin (Cambodia) that carries a C580Y mutation (K13^C580Y^), and the isogenic CamII_rev strain, which has the mutation reverted to the wild type (K13^WT^), restoring ART sensitivity. Mammalian orthologs of *Pf*ATG8, the LC3 (light chain 3), and of *Pf*ATG18, the WIPI1 (WD40 repeat protein interacting with phosphoinositides), participate in autophagy, with an increase in mRNA levels indicating autophagosome formation (53–55). The basal mRNA levels of *Pf*ATG8 and *Pf*ATG18 in synchronized rings as well as in young trophozoites of resistant and isogenic parasites were investigated by quantitative real-time PCR. Expression levels of *Pf*ATG8 in the K13^C580Y^ parasites relative to those in K13^WT^ show upregulation of ∼1.5-fold and ∼3-fold in early rings and young trophozoites, respectively, and *Pf*ATG18 shows ∼5.5-fold and ∼5-fold upregulation in early rings and young trophozoites, respectively (Fig. 2A and B). Concomitantly, immunoblot analysis shows increase in the expression levels of *Pf*ATG8 and *Pf*ATG18 by ∼2-fold in both early rings and young trophozoites of the resistant strain (Fig. 2C and D; see also Fig. S3A and B in the supplemental material at https://www.jncasr.ac.in/faculty/surolia/research-highlights/supplemental-material-mbio00630-22) compared to its isogenic counterpart. As increases in WIPI1 as well as LC3-labeled autophagosome numbers are an indicator of induced autophagy (55), the numbers of *Pf*ATG18- and *Pf*ATG8-decorated autophagosome-like vesicles were analyzed in K13^WT^ and K13^C580Y^ parasites. K13^C580Y^ parasites, in both early rings and young trophozoites, display an ∼2-fold increase compared to K13^WT^ in the number of *Pf*ATG18-labeled puncta colocalizing with *Pf*ATG8 (see Fig. S3C and D). The increase in expression of *Pf*ATG8 and *Pf*ATG18 at both the transcript and protein levels, as well as their colocalization, indicates activation of the autophagy pathway in resistant parasites, regulating various mechanisms of ART resistance, and it is thus not just a reflection of the accumulation of autophagosome-like vesicles in the parasite cytoplasm.

Induced levels of PI3P have already been reported in ART resistance (27). Also, PI3P regulates autophagy in yeast and higher organisms (56–58). We therefore carried out studies to check whether increased levels of PI3P during resistance reflect an increase in parasite autophagy at the ring stage. Immunofluorescence analysis was used to quantify the number of *Pf*ATG8-, *Pf*ATG18-, and PI3P-decorated puncta in K13^WT^ and K13^C580Y^ ring-stage parasites. As expected, the number of PI3P-labeled puncta shows a significant increase in K13^C580Y^ parasites compared to the number in K13^WT^ parasites (Fig. 2E and H). A similar increase in the numbers of *Pf*ATG8- and *Pf*ATG18-decorated puncta is also seen in the resistant parasites (Fig. 2F, G, and H) compared to its isogenic one. Taken together, the increased expression levels of autophagy proteins during ART resistance demonstrates parasite autophagy as a survival mechanism.

**FIG 3:**
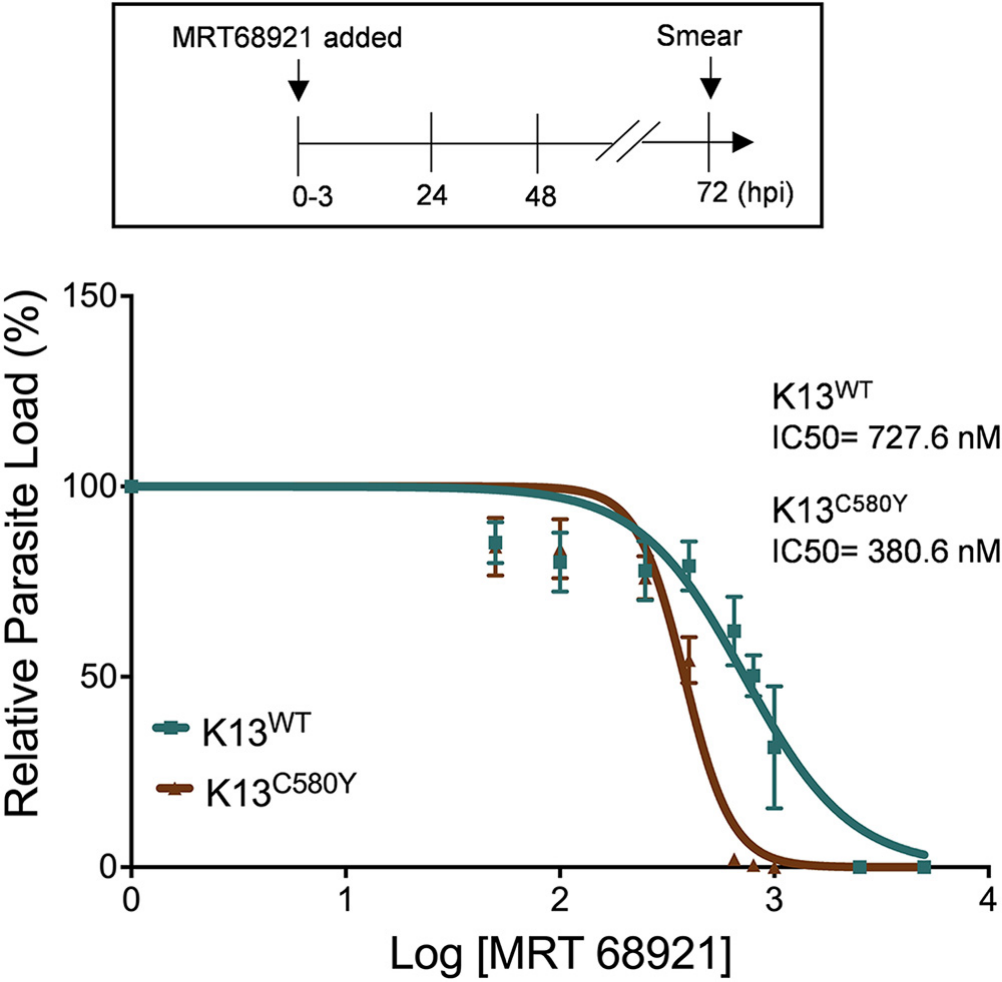
ART-resistant parasites are more sensitive to autophagy inhibition than their isogenic counterparts. The 0- to 3-hpi synchronized rings (K13^WT^ and K13^C580Y^) were treated with various concentrations of MRT68921 (as shown in the dose-response curve). The parasite load was monitored after 24 h, 48 h, and 72h. The dose-response curve shows the percentage of relative parasite load at 72 hpi in K13^WT^ (blue) and K13^C580Y^ (brown). *n* = 3 independent experiments. Data points are expressed as mean ± SEM.

To determine whether autophagy is essential for the survival of ART-resistant parasites, we compared the half-maximal inhibitory concentration (IC_50_) in K13^WT^ and K13^C580Y^ strains of MRT68921, a specific small molecule inhibitor of the human autophagy protein ULK1 (59). Tightly synchronized ring-stage parasites (0 to 3 hpi) from both strains (1% parasitemia) were incubated with various concentrations of the inhibitor for 72 h (Fig. 3). Giemsa-stained smears were used to monitor parasite morphology and invasion in the next cycle. K13^WT^ parasites display an IC_50_ value for MRT68921 of 727.6 nM, whereas it is only 380.6 nM in K13^C580Y^ parasites (Fig. 3). The decreased IC_50_ further emphasizes the importance of autophagy in the survival of ART-resistant parasites.

### Starvation induces parasite autophagy in an ART-resistant isolate, as well as in its isogenic isolate.

**FIG 4:**
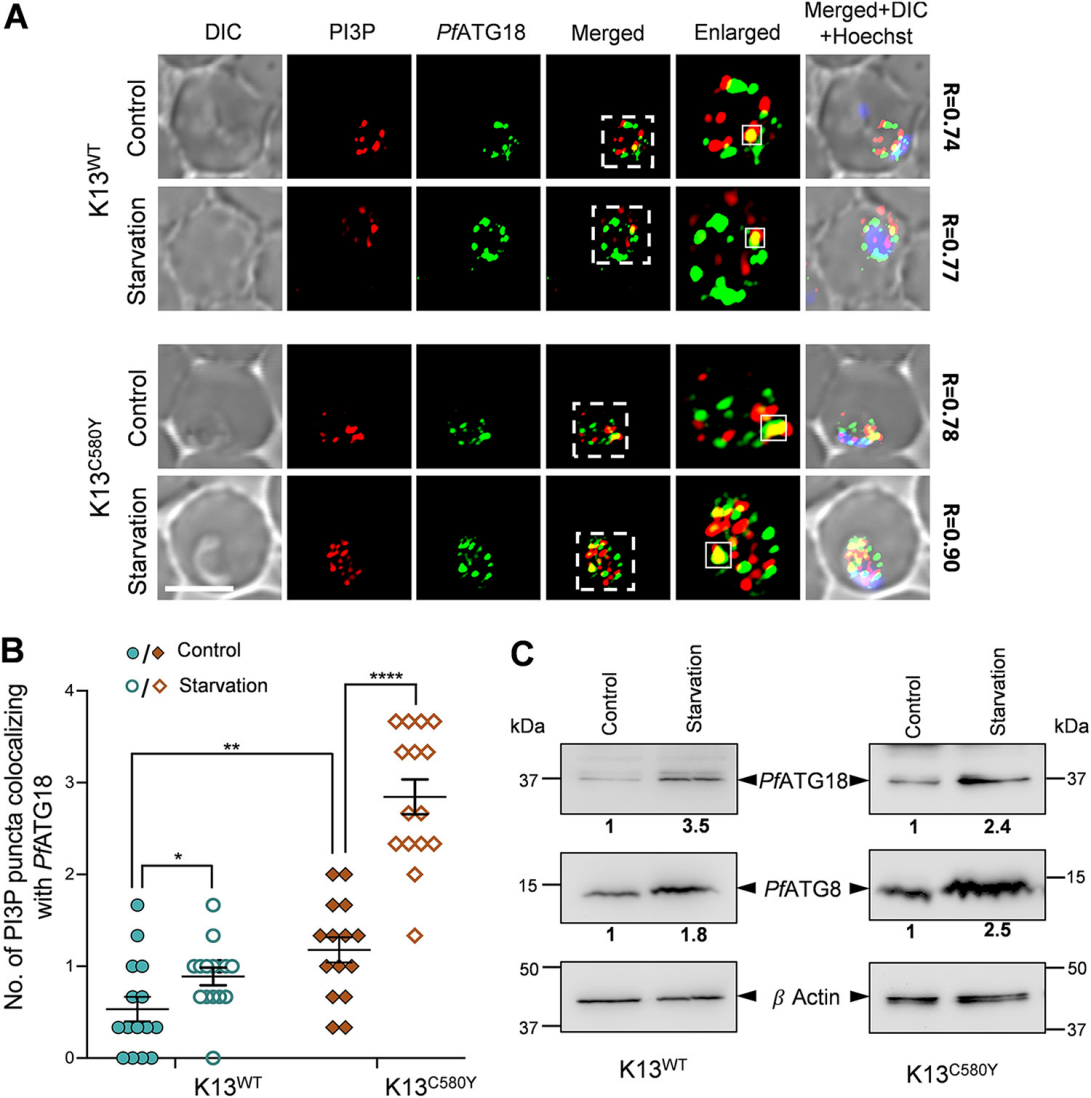
Starvation induces parasite autophagy in an ART-resistant isolate, as well as its isogenic isolate. K13^WT^ and K13^C580Y^ parasites were incubated with starvation medium for 1.5 h. (A) Immunofluorescence analysis of K13^WT^ (top two panels) and K13^C580Y^ (bottom two panels) parasites stained with anti-PI3P and anti-*Pf*ATG18 antibodies, showing colocalization of PI3P- with *Pf*ATG18-labeled puncta upon starvation. Regions within the dashed white lines are enlarged and placed next to the merged panel to represent colocalization. The extent of colocalization between *Pf*PI3P and *Pf*ATG18 is represented by the Pearson’s coefficient value (*R*) evaluated from PI3P (red) and *Pf*ATG18 (green) fluorescent signals within the region indicated with a white dashed square in the enlarged panel. The nucleus was stained using Hoechst stain. (B) Scatterplot representing the number of colocalizing PI3P- and *Pf*ATG18-labeled puncta in K13^WT^ and K13^C580Y^ parasites under control and starvation conditions. *N* = 15 parasites, *n* = 3 independent experiments. Bar, 5 μm. The data points are expressed as mean ± SEM. Statistical significance is quantified using unpaired Student’s *t* test; ****, *P* < 0.0001; **, *P* < 0.005; *, *P* < 0.05. (C) Effect of starvation on *Pf*ATG8 and *Pf*ATG18 protein expression levels. K13^WT^ and K13^C580Y^ parasite lysates were subjected to immunoblot analysis, and blots were probed with *Pf*ATG8 and *Pf*ATG18 antibodies. β-Actin was used as the loading control. *n* = 3 (K13^WT^) and 2 (K13^C580Y^) independent experiments. Fold difference, normalized with respect to the control, is shown below each blot.

As starvation induces parasite autophagy in the wild-type 3D7 strain (38, 40), the presence of a similar effect in an ART-resistant isolate and its isogenic parasites was investigated. An increase in the number of autophagosomes reflects upregulation of autophagy, and since both *Pf*ATG18 and PI3P are present on cytoplasmic vesicles of ∼200-nm diameter (38), we investigated the number of these autophagosome-like structures in the ART-resistant and isogenic parasites upon starvation by studying the colocalization of *Pf*ATG18 with PI3P-labeled puncta. Immunofluorescence analysis of synchronized rings (showing increased numbers of PI3P and *Pf*ATG18 puncta) incubated with starvation medium for 1.5 h reveals an increase in the number of *Pf*ATG18-labeled puncta colocalizing with PI3P upon starvation in both K13^WT^ and K13^C580Y^ parasites (Fig. 4A and B), with the increase in colocalization being more significant in K13^C580Y^ (Fig. 4B). Additionally, K13^C580Y^ parasites show an increase in *Pf*ATG18-labeled puncta colocalizing with PI3P at the basal levels compared levels in K13^WT^ (Fig. 4A and B). The increased binding of *Pf*ATG18 to PI3P further supports the interpretation that autophagy underlies various pathways of ART resistance. A similar increase is observed in the number of *Pf*ATG18- and PI3P-labeled puncta in K13^WT^ and K13^C580Y^ parasites upon starvation (see Fig. S4A to D in the supplemental material at https://www.jncasr.ac.in/faculty/surolia/research-highlights/supplemental-material-mbio00630-22). At the protein level, expression of *Pf*ATG8 and *Pf*ATG18 is also increased by ∼2-fold in both K13^WT^ and K13^C580Y^ parasites upon starvation (Fig. 4C; see also Fig. S4E and F). The immunoblot results corroborate our immunofluorescence analyses, demonstrating a functional autophagy pathway in both isogenic and resistant parasites that responds to autophagy induction under starvation conditions. Moreover, activation of autophagy is significantly higher in the resistant isolate compared to that in its isogenic one (Fig. 4B), which indicates reliance on autophagy for parasite fitness. The increased binding of *Pf*ATG18 with PI3P, along with the indued expression levels of parasite autophagy proteins upon starvation in the *Pf*K13^C580Y^ mutant, demonstrates that autophagy mediates the proteostasis mechanisms (increased PI3P vesiculation and upregulated UPR), as well as reduced hemoglobin endocytosis for survival of the parasite.

### *Pf*K13 as well as *Pf*ATG18 are trafficked to the food vacuole through hemoglobin-containing vesicles in the ART-resistant isolate.

**FIG 5:**
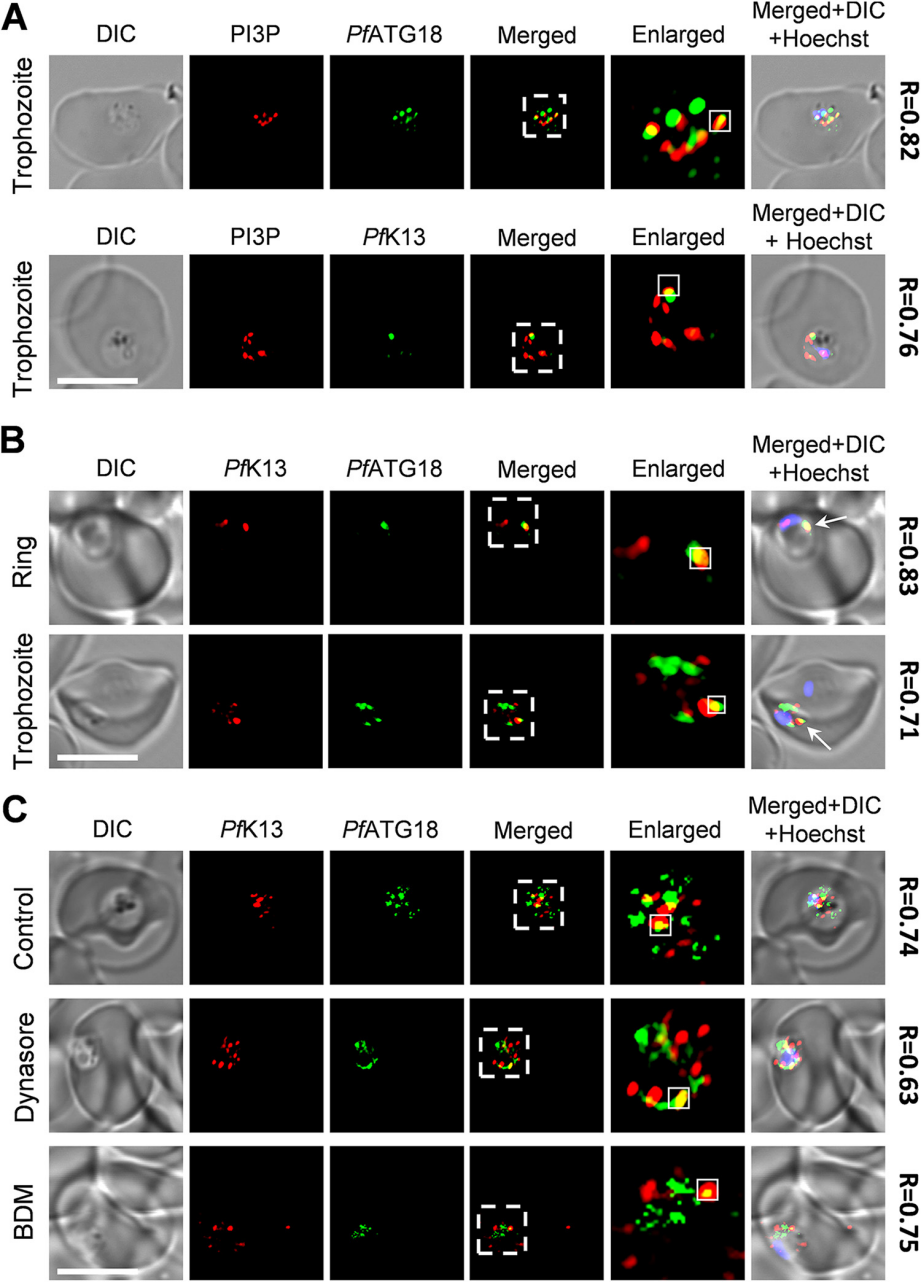
*Pf*K13 and *Pf*ATG18 are trafficked to the food vacuole (FV) through hemoglobin-containing vesicles (HCv) in the ART-resistant isolate. (A) Immunofluorescence analysis of K13^C580Y^ parasites stained with anti-PI3P, anti-*Pf*ATG18, and anti-*Pf*K13 antibodies showing colocalization of PI3P- with *Pf*ATG18- (top) and *Pf*K13 (bottom)-labeled puncta. (B) Immunofluorescence analysis of K13^C580Y^ parasites stained with anti-*Pf*ATG18 and anti-*Pf*K13 antibodies using the Zenon antibody labeling system, showing colocalization of *Pf*K13- with *Pf*ATG18-labeled puncta in rings (top) and young trophozoites (bottom). Rings show more colocalization toward the parasite boundary (white arrow), while trophozoites show more near the parasite FV (white arrow). *N* = 15 parasites, *n* = 3 independent experiments. Bar, 5 μm. (C) *Pf*ATG18 and *Pf*K13 are transported to parasite FV through the hemoglobin trafficking pathway. Localization of *Pf*ATG18- and *Pf*K13-labeled puncta upon incubation of parasites with hemoglobin trafficking inhibitors, 200 μM Dynasore, and 25 mM 2,3-butanedione monoxime (BDM) was assessed using immunofluorescence analysis. K13^C580Y^ parasites were stained with anti-*Pf*ATG18 and anti-*Pf*K13 antibodies using the Zenon antibody labeling system. Incubation of parasites with inhibitors led to colocalization of *Pf*ATG18 and *Pf*K13 near the parasite periphery. *N* = 10 parasites, *n* = 2 independent experiments. Bar, 5 μm. Regions within the dashed white lines are enlarged and placed next to the merged panel to represent colocalization. The extent of colocalization is represented using the Pearson’s coefficient value (*R*) evaluated from the PI3P and *Pf*K13 (red) and *Pf*ATG18 (green) fluorescent signals within the white square region in the enlarged panel. The nucleus was stained using Hoechst stain.

The endocytosis pathway in P. falciparum plays a crucial role in the host cell cytosol uptake (HCCU) involving the ingestion of host hemoglobin by the parasite (60). Recent reports highlight the importance of *Pf*K13 in regulating endocytosis and thus controlling the amount of hemoglobin uptake by the peripherally localized parasite vesicle “cytostomes” (34, 61, 62). These vesicles are formed due to the parasite plasma membrane invaginations that deliver host hemoglobin to the parasite food vacuole (63, 64). *Pf*K13 localizes to various subcellular compartments in the parasite, including ER-PI3P vesicles, the apicoplast, the food vacuole (FV), and the collar region of cytostomes (26, 27, 34, 62). However, there is no experimental evidence suggesting the presence of *Pf*K13 on hemoglobin-containing vesicles (HCv) which are discrete vesicles transporting host derived hemoglobin to the parasite FV (65). Our previous report indicates that trafficking of *Pf*ATG18 to the FV is via HCv and is mediated by the interaction of *Pf*ATG18 with PI3P (38). To investigate whether *Pf*K13 cotrafficks with *Pf*ATG18 to the FV in the ART-resistant K13^C580Y^ parasites, immunofluorescence analysis with young trophozoites was carried out. The cysteine protease falcipain-2 (*Pf*FP2) is trafficked to the FV via HCv (66) and was thus used to label the vesicles. *Pf*ATG18 partially colocalizes with both PI3P (Fig. 5A, top) and *Pf*FP2 (see Fig. S5A in the supplemental material at https://www.jncasr.ac.in/faculty/surolia/research-highlights/supplemental-material-mbio00630-22, top) in the resistant K13^C580Y^ parasites, similarly to observations in wild-type 3D7 parasites (38). Also, partial colocalization of *Pf*K13 with PI3P (Fig. 5A, bottom) and PfFP2 (see Fig. S5A, bottom) is observed near the parasite periphery as well as in the cytoplasm, indicating the presence of *Pf*K13 and *Pf*ATG18 on HCv. To confirm this, immunofluorescence analysis with *Pf*ATG18 and *Pf*K13 antibodies was carried out at ring and young trophozoite stages of the K13^C580Y^ parasites. Our data show colocalization of *Pf*K13-labeled with *Pf*ATG18-labeled puncta near the parasite periphery in rings, suggesting their copresence on cytostome-like structures (Fig. 5B, top). Presence of *Pf*ATG18-labeled and *Pf*K13-labeled puncta near the ring periphery is confirmed by labeling the parasite vacuolar membrane (PVM) with a marker protein (67), *Pf*PTEX-150 (see Fig. S5B). Parallelly, *Pf*K13-labeled puncta colocalize with *Pf*ATG18-labeled puncta near the FV in trophozoites (Fig. 5B, bottom) as implied by their localization near the parasite hemozoin, which we propose occurs due to their enhanced cotrafficking via HCv to the FV in mature parasite stages.

Trafficking of HCv to the FV is assisted by the parasite actin-myosin motor system (68). To further support that *Pf*ATG18 and *Pf*K13 proteins are transported to the FV via the HCv, young trophozoites were treated with hemoglobin-trafficking inhibitors (2,3-butanedione monoxime [BDM] and Dynasore) which block HCv transport to the FV. Parasites were incubated with myosin ATPase inhibitor BDM and Dynasore which inhibits the GTPase activity of dynamin, both causing accumulation of HCv to the parasite periphery (68). Colocalization of *Pf*K13- with *Pf*ATG18-labeled puncta is observed more toward the parasite boundary upon incubating parasites with these inhibitors compared to the near-FV localization seen in control (Fig. 5C). We propose interactions between *Pf*K13 and *Pf*ATG18, which is a key finding for the association of a parasite autophagy protein with the resistant marker *Pf*K13 in ART resistance.

### Co-occurrence of *Pf*K13 and *Pf*ATG18 mutations in the field samples of P. falciparum.

The mutation in *Pf*ATG18 has been reported to have strong selection under ART resistance (18, 44). Interestingly, *Pf*ATG18 T38I confers fitness advantage to the parasite by enabling a higher growth rate under nutrient-limiting conditions and upon ART resistance (44). The wild-type *Pf*ATG18 at T38 is believed to be phosphorylated (44). While phosphorylation of ATG18 in yeast is shown to control organellar dynamics, its dephosphorylated form has increased affinity for the phosphoinositide, necessary for association with ATG18 and the membranes (69). On the other hand, the *Pf*K13 mutations (C580Y, Y493H, and R539T) cause low growth of the parasite due to reduced hemoglobin endocytosis (34, 70). As ART gets activated by the degradation product (heme) of the host hemoglobin, the reduced hemoglobin endocytosis in ART resistance inhibits activation of ART, rendering the parasite drug resistant. Thus, the K13 mutations and *Pf*ATG18 T38I mutation exhibit contrasting but complementary/compensatory characteristics. However, the frequency of association between *Pf*K13 and *Pf*ATG18 mutations, which mediates homeostasis, has not yet been demonstrated.

**FIG 6:**
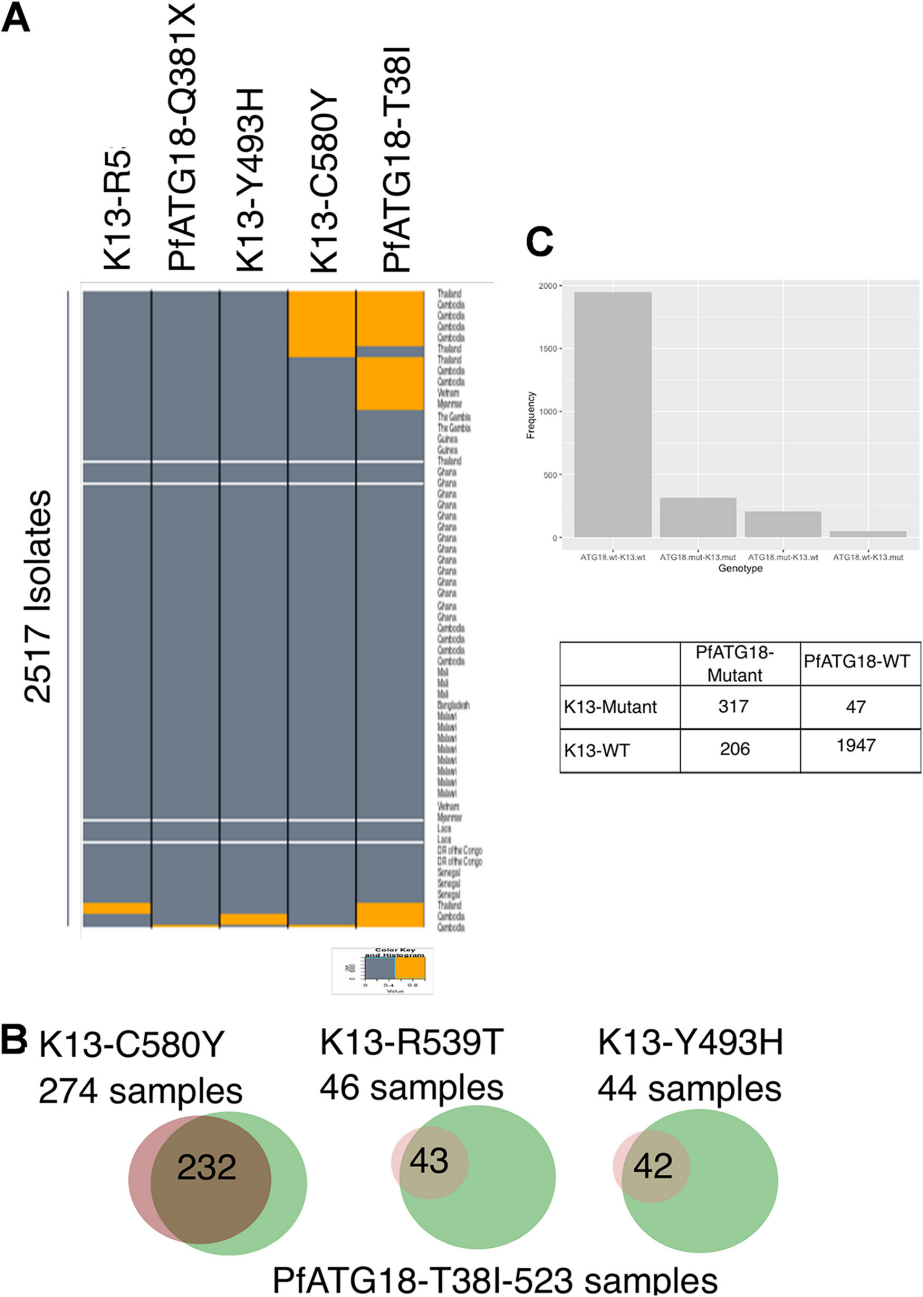
Co-occurrence of *Pf*K13 and *Pf*ATG18 mutations in the field samples of P. falciparum. (A) Plot showing coexistence of various K13 mutations (C580Y, R539T, and Y493H) and *Pf*ATG18 mutations (T38I and *381Q) in 2,517 genome samples. (B) Co-occurrence of Kelch13 mutations (C580Y, R539T, and Y493H) and *Pf*ATG18 mutation (T38I) in 2,517 genome samples analyzed from 18 different geographical locations. The majority of K13 mutations co-occur with *Pf*ATG18-T38I mutation, as follows: 84.6% with C580Y, 93.4% with R539T, and 95.4% with Y493H. (C) Frequency plot and contingency table listing the genotype and frequency of mutations in *Pf*ATG18 and Kelch13 genes.

In this study, we have analyzed 2,517 genomes of P. falciparum from Southeast Asia and Africa. We identified six nonsynonymous mutations in *Pf*Atg18, including a stop codon mutation, *381Q (Table 1). We find T38I mutation in 523 samples out of 2,517 total samples (Fig. 6A). Interestingly, we observe that the *Pf*ATG18-K13 double mutant genotype (where the K13 mutant may be any of C580Y, R539T, or Y493H) is the second most common genotype over these two loci. The frequency of T38I-ATG18 mutation is 84.6% in C580Y, 93.4% in R539T, 95.4% in Y493H in K13 mutations (Fig. 6B). We find that most K13 mutants also possess an ATG18 mutation (317 out of 364), indicating that the two mutations may be associated in their occurrence (Fig. 6C). In order to statistically examine this possibility, we calculated the observed probability of finding an ATG18 mutation given a K13-mutant background as being ∼87% (using equation 1). We similarly calculated the expected probability of ATG18.mut given K13.mut as ∼20.8% by substituting equation 2 into equation 1.

(1) P(ATG18.mut | K13.mut) = P(ATG18.mut ∩ K13.mut) ÷ P(K13.mut)

(2) P(ATG18.mut ∩ K13.mut) = P(ATG18.mut) × P(K13.mut) 

**TABLE 1:** List of nonsynonymous mutations identified in gene PF3D7_1343700 (encoding the autophagy-related protein *Pf*ATG18)

Nonsynonymous mutation (PF3D7_1343700)	Genomic coordinate
*381Q(A/G)	496333
K301N (T/A)	496571
N298S (T/C)	496581
A295V (G/A)	496590
K275T (T/G)	496650
T38I (G/A)	497461

If ATG18 mutations and K13 mutations were independent events, one would intuitively expect equation 1 to reduce to the following:

(3) P(ATG18.mut | K13.mut)= P(ATG18.mut)

However, the equation shown above does not hold for our observations, further suggesting that the two mutations co-occur. In order to statistically examine this possibility, we performed a χ^2^ test for independence in R, testing the standard null hypothesis that the two mutations are not associated using a contingency table (Fig. 6C). We also performed a chi-squared test with a Yates’ correction and one with 2,000 and 1,000,000 iterations of Monte Carlo simulation (codes provided in the supplemental material at https://www.jncasr.ac.in/faculty/surolia/research-highlights/supplemental-material-mbio00630-22). We found that they co-occur to a significant degree (*P* < 0.001). Thus, our data demonstrate co-occurrence and coassociation of *Pf*K13 C580Y and *Pf*ATG18 T38I mutations in field isolates of P. falciparum, reaffirming the association between these two proteins in ART resistance.

## DISCUSSION

Our studies demonstrate that stress-induced autophagy underpins various mechanisms of ART resistance and advance the understanding of the two recently proposed mechanisms of ART resistance involving induced ER and cytoplasmic proteostasis mechanisms mitigating ART-mediated proteopathy (26, 27), as well as the *Pf*K13 C580Y mutation-associated reduced hemoglobin endocytosis pathway (34).

We show that ART-induced UPR results in increased autophagy in the parasite (Fig. 1), which may render a fitness advantage to it during resistance (Fig. 3). Our results on the expansion of parasite ER, as well as the increased phosphorylation of *Pf*eIF2α upon incubation with DHA (see Fig. S1 in the supplemental material at https://www.jncasr.ac.in/faculty/surolia/research-highlights/supplemental-material-mbio00630-22), is consistent with previous reports showing ART induced ER stress responses (33, 46). In the absence of the conventional UPR transcription factors, ATF6 and XBP1 (71), the parasite needs to manage excess protein misfolding under ER stress conditions. The increase in the number of *Pf*ATG8-labeled autophagosome-like vesicles, as well as the expression levels of *Pf*ATG8 and *Pf*ATG18 upon DHA treatment, demonstrate induction of the parasite autophagy pathway in response to ER stress and the consequent UPR (Fig. 1). Also, pharmacological inhibition of the PERK-*Pf*eIF2α-driven UPR pathway leads to a reduction in the expression of autophagy proteins, suggesting that their activation is through the UPR/PERK pathway in the parasite (Fig. 1). Although ART reduces global protein synthesis through phosphorylation of *Pf*eIF2α (46), increased expression levels of autophagy proteins signify the importance of autophagy in alleviating the effect of protein misfolding as a result of ART exposure.

Furthermore, the observation of increased baseline expression of *Pf*ATG8 and *Pf*ATG18 (Fig. 2A to D), as well as colocalization of *Pf*ATG18-labeled with *Pf*ATG8-labeled puncta (see Fig. S3C and D in the supplemental material at https://www.jncasr.ac.in/faculty/surolia/research-highlights/supplemental-material-mbio00630-22) in ART-resistant parasites during the early ring and young trophozoite stages, compared to their isogenic counterpart, demonstrates that parasite autophagy governs the regulation of various proteostasis mechanisms of ART resistance. We propose that, since host hemoglobin endocytosis is decreased due to the C580Y mutation (34), autophagy rescues the parasites from nutrient-limiting conditions and is therefore responsible for survival through delayed progression to the trophozoite stage. This is reflected in the higher expression levels of *Pf*ATG8 and *Pf*ATG18 (Fig. 2C) combined with the increased numbers of *Pf*ATG8-, *Pf*ATG18-, and PI3P-decorated vesicles (Fig. 2E to H) in the early rings of ART-resistant parasites compared those of its isogenic strain. As the parasite stage progresses toward young trophozoites, heme accumulates in the FV due to increased hemoglobin uptake and synthesis (65, 72), culminating in oxidative stress, which promotes autophagy (73). Enhanced ER-PI3P vesiculation disseminating the *Pf*BiP chaperone (27, 28) also leads to increased expression levels of P. falciparum autophagy proteins, which underlie the proteostasis mechanisms in both the ER and the cytoplasm. Together, our results establish the role of autophagy in mediating various mechanisms of ART resistance. Additionally, modulation of autophagy by starvation or pharmacological inhibition by MRT68921 has a more profound effect on the resistant parasites than on the isogenic ones (Fig. 3 and 4). The increased number of PI3P-bound *Pf*ATG18 vesicles in the *P*K13 C580Y mutant strain compared to those in its isogenic strain at the basal level itself indicates that autophagy is initiated in resistant parasites even in the absence of an external stressor (Fig. 4A and B). The increased PI3P vesiculation reported during resistance may be directly related to the induction of autophagy proteins, which promotes *Pf*ATG18 binding to these vesicles and recruiting other autophagy proteins, which is necessary for the formation of autophagosome-like structures in the parasite. The increased number of these vesicles in both resistant and isogenic parasites upon autophagy induction by starvation (Fig. 4A and B) further confirms a conserved role of *Pf*ATG18 in parasite autophagy and demonstrates a functional autophagy pathway that responds to autophagy induction under starvation. Additionally, expression of autophagy proteins is much higher in the resistant isolate than in its isogenic isolate, showing the parasite’s dependence on autophagy for fitness.

Furthermore, when we examined whether the *Pf*K13-decorated vesicles carrying hemoglobin are the same subcellular compartments on which *Pf*ATG18 and PI3P colocalize, a partial colocalization of *Pf*K13, PI3P, and *Pf*ATG18 on these vesicles (which are HCv) was observed (Fig. 5; see also Fig. S5A in the supplemental material at https://www.jncasr.ac.in/faculty/surolia/research-highlights/supplemental-material-mbio00630-22). Other routes for hemoglobin delivery to the FV are also known, including processes involving phagotrophs and the microtubule-assisted cytostomal tubes (60, 65), which explains the partial colocalization of *Pf*K13-*Pf*ATG18-PI3P with *Pf*FP2-decorated vesicles. We speculate that the increased colocalization of *Pf*K13 and *Pf*ATG18 toward the parasite periphery at the ring stage, while they are localized close to the FV in trophozoites (Fig. 5B), is due to their enhanced cotrafficking to the FV in the trophozoite stage.

ART resistance is a complex interplay between K13 mutations and the genetic background, which either directly imparts ART resistance or augments a fitness advantage to the parasites (74). Furthermore, mutations in *Pf*K13 also contribute to the stress responses experienced by resistant parasites due to the decrease in availability of host hemoglobin and resulting in upregulation of parasite autophagy. However, ART resistance is not limited to *Pf*K13 and autophagy proteins alone but is associated with the genetic background and expression of chaperones reported in *in vitro* cultures, as well as the UPR pathway involving Plasmodium reactive oxidative stress complex (PROSC) and TCP-1 ring complex (TRiC) chaperone complexes in resistant clinical isolates *in vivo* (28, 75). These factors, along with the UPR-mediated increased autophagy and enhanced *Pf*eIF2α phosphorylation demonstrated in this study, further advances autophagy as the key player of proteostasis mechanisms of ART resistance.

Coassociation of the *Pf*K13 C580Y mutation with the *Pf*ATG18 T38I mutation in 85% of 2,517 field isolates (Fig. 6) found in our single-nucleotide polymorphism (SNP) data analysis further validates the conclusions of our studies. As the *Pf*ATG18 T38I mutation provides a fitness advantage (44), and parasites with *Pf*K13 C580Y mutations experience decreased nutrient availability, we propose that the copresence of these two mutations imparts a fitness advantage to the parasite by promoting autophagy that serves as an additional source of nutrients, supporting parasite growth during ART-mediated proteotoxicity and stress.

**FIG 7:**
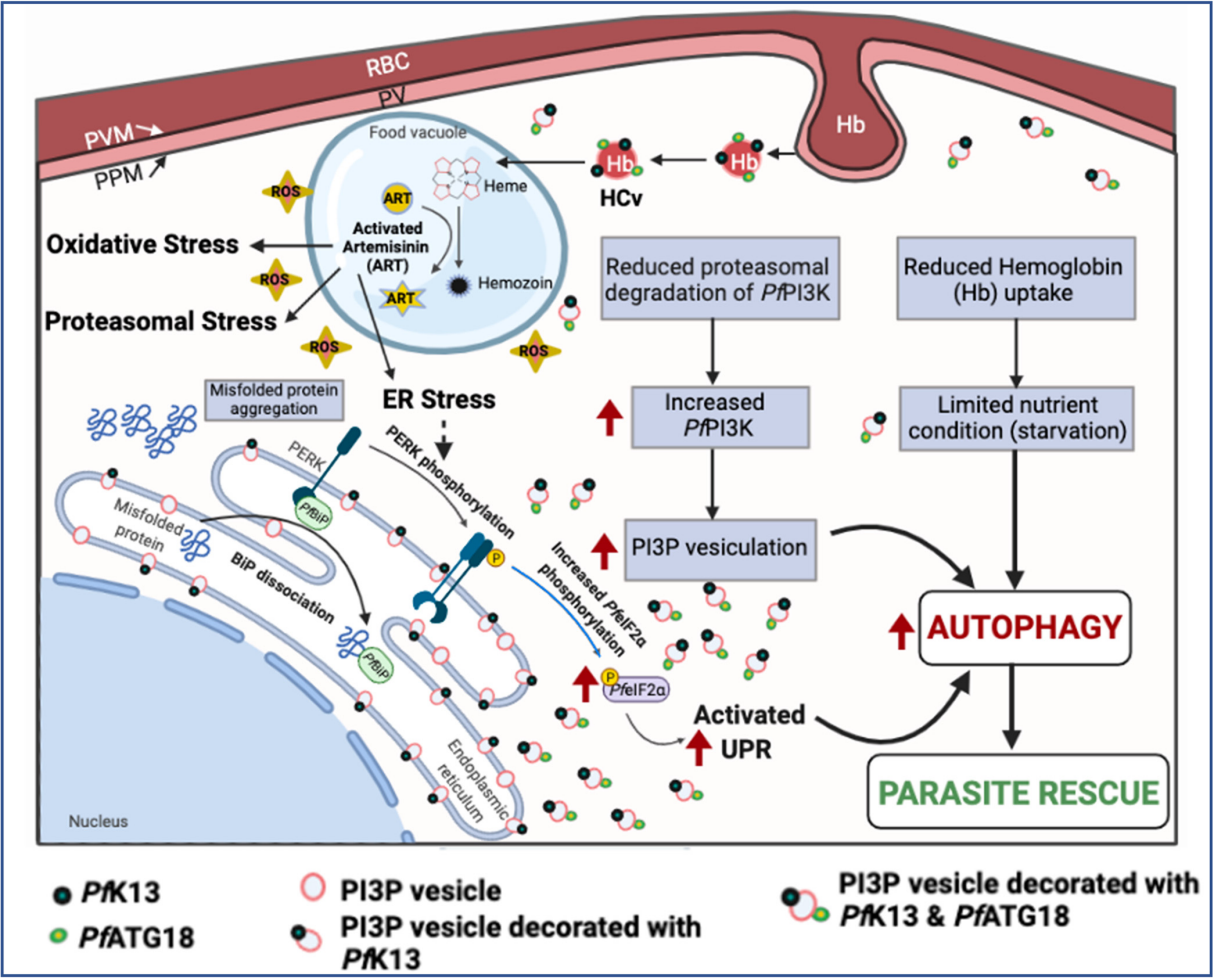
Schematic representation for role of autophagy in proteostasis mechanisms of ART resistance in P. falciparum. Activated ART generates reactive oxygen species (ROS) and leads to alkylation and misfolding of proteins, causing activation of the stress response pathways. The major source of amino acid supply, host hemoglobin endocytosis, is diminished at the ring stage in *Pf*K13 mutants, reducing ART activation and decreasing protein misfolding. The reduced hemoglobin uptake in *Pf*K13 mutants results in limited nutrient conditions that induces autophagy. Also, the decrease in PI3K ubiquitination and degradation leads to increased PI3P vesiculation, which induces the parasite autophagy pathway. Our results reveal that *Pf*K13 mutants have increased levels of autophagy proteins, indicating the role of autophagy in the survival of the resistant parasites.

Our findings collectively establish a unifying role for autophagy as a cellular process interlinking various mechanisms of ART resistance (Fig. 7). In addition, the inhibition of ART-resistant parasites by a very specific autophagy inhibitor, MRT68921, identifies this pathway as a novel target, which needs further efforts to develop new and more effective therapeutics.

## MATERIALS AND METHODS

### Materials.

RPMI 1640, HEPES, sodium bicarbonate (NaHCO_3_), hypoxanthine, d-sorbitol, MRT68921, GSK2606414, 2,3-dutanedione monoxime, Hoechst 33258, phenylmethylsulfonyl fluoride (PMSF), TRIzol reagent, DNase I, and oligonucleotides were purchased from Sigma-Aldrich, USA; RPMI 1640 without amino acids from HyClone Laboratories, Inc., USA; AlbuMax II from Gibco; dihydroartemisinin from AK Scientific; Dynasore hydrate and polyvinylidene difluoride (PVDF) membranes from Millipore Sigma, USA; WR99210 from Jacobus Pharmaceutical Co., USA; paraformaldehyde (EM grade) from ProSciTech, Australia; Vectashield from Vector Laboratories; protein inhibitor cocktail from Roche; Clarity Western ECL substrate and iTaq UniverSYBR green from Bio-Rad; Zenon Alexa Fluor 488 rabbit IgG labeling kit from Thermo Fisher Scientific; and restriction enzymes, M-MuLV reverse transcriptase and oligo(dT) primers from New England BioLabs, USA. The following commercially available antibodies were obtained: anti-PI3P antibody by Echelon Biosciences; anti-phospho-eIF2α by Cell Signaling; anti-actin antibody and anti-green fluorescent protein (GFP) antibody by Sigma-Aldrich; anti-KDEL antibody by Abcam; anti-rabbit and anti-mouse Alexa Fluor 488 and Alexa Fluor 568 by Invitrogen; and anti-rabbit-horseradish peroxidase (HRP) by Bio-Rad. The following Plasmodium falciparum-specific antibodies were custom generated: anti-*Pf*ATG18 by GenScript, USA; anti-eIF2α by Bioklone, India; anti-*Pf*ATG8 and anti-*Pf*PTEX-150 by Genemed Biotechnologies, Inc. Anti-BiP antibody was obtained by MR4, Bei Resources; anti-K13 and anti-falcipain-2 antibodies were kind gifts from Souvik Bhattacharya (JNU, New Delhi, India) and Asif Mohammed (ICGEB, New Delhi, India), respectively.

### P. falciparum cultures.

Parasite cultures were maintained by the candle jar method (76) and cultured in O-positive (O+) human red blood cells (RBCs) using complete RPMI 1640 supplemented with 25 mM HEPES, 0.2% NaHCO_3_, 0.5 mM hypoxanthine, 0.5% (wt/vol) AlbuMax II, and 5% (vol/vol) heat-inactivated O+ human serum. For starvation experiments, cultures were maintained in RPMI 1640 without amino acids or serum for 1.5 h. Subculturing was done after every 48 h. Cultures were synchronized by treatment with 5% d-sorbitol (77). For isolation of parasites, RBCs were lysed using 0.03% (wt/vol) saponin incubated for 10 min on ice, followed by centrifugation. The pellet obtained was washed using cold phosphate-buffered saline (PBS; pH 7.4, 1×).

### Plasmid construction and transfection.

PCR amplification of full-length *Pf*Sec62 (stop codon omitted) from 3D7 cDNA was done using the primers 5′-GGCCGGTACCATGAGTA ACAGAATGGAGGAATTAG-3′ (KpnI site underlined) and 5′-GCGCCCTAGGATTGT CTGATTTATCAAAACATGCTTC-3′ (AvrII site underlined) and digested with restriction enzymes KpnI and AvrII, followed by ligation into the pARL1-a vector (78) at the same sites. The pARL1-a vector contains a C-terminal GFP tag, a promoter region for *Pf*CRT, and human dihydrofolate reductase (hDHFR) as the drug selection cassette. The resultant plasmid was episomally expressed in the parasites.

Transfection in P. falciparum 3D7 parasites was done as previously described (79). Briefly, synchronized ring-stage parasites with 5% parasitemia were mixed with CytoMix (0.15 mM CaCl_2_, 120 mM KCl, 2 mM EGTA, 10 mM K_2_HPO_4_/KH_2_PO_4_, 5 mM MgCl_2_, and 25 mM HEPES, [pH 7.6]) containing 100 μg of *Pf*Sec62-GFP cloned in a pARL1-a plasmid. This was electroporated using a GenePulser II instrument (0.31 kV, 960 μF) and resuspended immediately in complete medium containing fresh RBCs. Subsequently, the culture was maintained in 2.5 nM WR99210.

### Parasite treatment with small molecule inhibitors.

Early rings and young trophozoites were treated with 700 nM DHA for the indicated periods of time. Young trophozoites were treated with 2.5 μM MRT68921 for 1.5 h, 30 μM GSK2606414 for 2.5 h, 200 μM Dynasore hydrate for 4 h, and 25 mM 2,3-butanedione monoxime (BME) for 1 h.

### Immunofluorescence assay.

The assay was performed as described previously (80). Parasite-infected RBCs were washed three times with 1× PBS and fixed at room temperature for 30 min using 4% paraformaldehyde and 0.0075% glutaraldehyde in PBS. After fixation, cells were washed three times, followed by permeabilization at room temperature for 10 min using 0.1% Triton X-100–PBS. Blocking was done at 4°C for 1 h using 3% bovine serum albumin (BSA)-PBS, followed by incubation with the primary antibody in 3% BSA-PBS at room temperature for 1 h. Primary antibodies were used at the following dilutions: rabbit anti-*Pf*ATG18 (1:400), rabbit anti-*Pf*ATG8 (1:600), mouse anti-PI3P (1:500), rabbit anti-falcipain-2 (1:400), and rabbit anti-K13 (1:400). This was followed by three washes and incubation with secondary antibodies using 3% BSA-PBS at room temperature for 1 h. Alexa Fluor 568 and 488 conjugated to goat anti-mouse (1:200) and goat anti-rabbit (1:200) antibodies were used as secondary antibodies. After staining the nucleus with Hoechst 33258, cells were mixed with Vectashield and then mounted over glass slides and observed under a confocal microscope. The mean ± standard error of mean (SEM) from each experiment was plotted using Prism 9 software (GraphPad; La Jolla, CA).

### Microscopy.

For visualizing GFP signals in parasites expressing *Pf*Sec62-GFP, samples were incubated with Hoechst 33342 for 15 min and washed three times with PBS, followed by mounting over glass slides. Signals were observed using the LSM 700 (Carl Zeiss, Germany) confocal microscope using a 488-nm laser and the 63×, 1.4 numerical aperture (NA) oil objective. Immunofluorescence signals from fixed samples were observed using LSM 700 and Airyscan confocal microscopes (Zeiss). Image processing was performed with Imaris (Bitplane, Zurich, Switzerland) and Zen (blue edition). The distance was quantified using the line profile in Zen software and the extent of colocalization was measured by Pearson’s coefficient (*R*).

### Zenon labeling.

For coimmunolocalization, Zenon labeling was used. The first primary antibody labeling was performed using an immunofluorescence assay protocol as described previously (80). The second primary antibody was conjugated with Zenon rabbit IgG 488 in a 6:1 molar ratio (supplier recommendation). Samples were incubated with this complex for 40 min, followed by imaging within 1 h to avoid dissociation of the complex.

### Immunoblot analysis.

The parasite pellet, obtained by saponin treatment as described previously (77), was lysed in buffer containing 1% Triton X-100 in 1× PBS, 1× complete protein inhibitor cocktail, and 1 mM PMSF, followed by passing through a syringe 5 to 10 times. Protein extract obtained as a supernatant by high-speed centrifugation was then boiled for 10 min followed by protein separation on an SDS-PAGE gel. The proteins were transferred to a Immobilon PVDF membrane and specific proteins were detected by these antibodies (raised in rabbits), anti-*Pf*ATG8 (1:4,000), anti-*Pf*ATG18 (1:4,000), anti-eIF2α (1:500), anti-BiP (1:2,500), anti-phospho-eIF2α (1:500), and anti-eIF2α (1:500). β-Actin, used as a loading control, was detected by rabbit anti-actin antibodies (1:5,000). Horseradish peroxidase-conjugated goat anti-rabbit antibody (1:3,000) was used as the secondary antibody. Immunoblot signals were developed by Clarity Western ECL substrate and imaged using the ChemiDoc imaging system (Bio-Rad). Densitometry analysis of the specific protein bands was performed using ImageJ software. The mean ± SEM from each experiment was plotted using GraphPad.

### Quantitative real-time PCR.

The parasite (K13^C580Y^ and K13^WT^) pellets were obtained by the saponin treatment as described previously (77), and total RNA was extracted using TRIzol reagent following the manufacturer’s protocol. The isolated RNA was then treated with RNase-free DNase I to remove any genomic DNA contamination. A 1-μg aliquot of the extracted RNA was then used for cDNA synthesis using the M-MuLV reverse transcriptase and oligo(dT) primers in a 20-μL reaction mixture. Another set of cDNA synthesis reactions was set up without the reverse transcriptase enzyme as a negative control. Quantitative real-time PCR with 10 ng of cDNA was used to amplify the genes of interest, *Pf*atg8 and *Pf*atg18. The primer sequences used to amplify regions in the genes were as follows: for *Pf*atg8, 5′-CATCAACATATTAATCAAAGTGCATATGG-3′ and 5′-AAATCTTGCATTAACAATCCTGTTTTAGG-3′; for *Pf*atg18, 5′-ATATGTAAAGGAAAAAATGTATCCCC-3′ and 5′-GCAAAACTCCATTCACTATTTAAATAC-3′; and for β-actin, 5′-CCATGAAAATTAAAGTTGTTGCACCAC-3′ and 5′-TTGGTCCTGATTCATCGTATTCCTC-3′. A SYBR green-based fluorescent tag was used for DNA labeling in a CFX96 Touch real-time PCR detection system (Bio-Rad, USA), and the abundance of each transcript was determined using the threshold cycle (*C_T_*). The housekeeping gene β-actin was used to normalize the gene expression and fold differences in the expression of these genes were calculated using the 2^−ΔΔ^*^CT^* method (81). The mean ± SEM from the experiment was plotted using GraphPad.

### Growth inhibition assay.

To assess the effect of MRT68921 on P. falciparum growth, parasites were tightly synchronized by sorbitol treatment. Ring-stage K13^WT^ and K13^C580Y^ parasites with 1% parasitemia and 3% hematocrit were incubated with various concentrations of MRT68921 (5,000 nM, 2,500 nM, 1,000 nM, 800 nM, 650 nM, 400 nM, 250 nM, 100 nM, and 50 nM in triplicate) in a 96-well cell culture plate. Thin blood smears were made from each well after ∼72 hpi and were stained with Giemsa for microscopic analysis. The percentage of parasites in the next life cycle was calculated for each concentration of MRT68921 and was plotted as a function of MRT68921 concentration using GraphPad and the half-maximal inhibitory concentration (IC_50_) was estimated using the dose-response function of GraphPad.

### Genome analysis.

To analyze the coexistence of SNPs in gene PF3D7_1012900 (*Pf*ATG18) with SNPs of Kelch gene PF3D7_1343700 (kelch protein K13), we downloaded the SNP data from *Pf*3K MalariaGEN (pilot data release 4; https://www.malariagen.net/data/pf3k-pilot-data-release-4). There were 2,640 samples for the K13 gene from 18 different geographical locations, primarily from African (*n* = 884) and Asian (*n* = 1,627) subcontinents and lab strains (*n* = 129). We identified 3 SNPs (C580Y, Y493H, and R539T) that are known as potential biomarkers for ART resistance and identified the presence of those mutations in all of the samples using in-house R scripts. Variant calling was done for 2,517 samples for PF3D7_1012900 (*Pf*ATG18), excluding the lab strains (mixed lab strain and genetic crosses). To maintain the homogeneity of the data, only 2,517 samples were compared for further analysis. The plots were generated using R scripts.

## References

[1] World Health Organization. 2021. World malaria report 2021. World Health Organization, Geneva, Switzerland.

[2] Balint GA. 2001. Artemisinin and its derivatives: an important new class of antimalarial agents. Pharmacol Ther 90:261–265. doi:10.1016/s0163-7258(01)00140-1. 11578659

[3] Woodrow CJ, Haynes RK, Krishna S. 2005. Artemisinins. Postgrad Med J 81:71–78. doi:10.1136/pgmj.2004.028399. 15701735 PMC1743191

[4] Chawira AN, Warhurst DC. 1987. The effect of artemisinin combined with standard antimalarials against chloroquine-sensitive and chloroquine-resistant strains of *Plasmodium falciparum in vitro*. J Trop Med Hyg 90:1–8. 3546715

[5] Aweeka FT, German PI. 2008. Clinical pharmacology of artemisinin-based combination therapies. Clin Pharmacokinet 47:91–102. doi:10.2165/00003088-200847020-00002. 18193915

[6] Cui L, Su X. 2009. Discovery, mechanisms of action and combination therapy of artemisinin. Expert Rev Anti Infect Ther 7:999–1013. doi:10.1586/eri.09.68. 19803708 PMC2778258

[7] Noedl H, Se Y, Schaecher K, Smith BL, Socheat D, Fukuda MM, Artemisinin Resistance in Cambodia 1 (ARC1) Study Consortium. 2008. Evidence of artemisinin-resistant malaria in western Cambodia. N Engl J Med 359:2619–2620. doi:10.1056/NEJMc0805011. 19064625

[8] Dondorp AM, Nosten F, Yi P, Das D, Phyo AP, Tarning J, Lwin KM, Ariey F, Hanpithakpong W, Lee SJ, Ringwald P, Silamut K, Imwong M, Chotivanich K, Lim P, Herdman T, An SS, Yeung S, Singhasivanon P, Day NPJ, Lindegardh N, Socheat D, White NJ. 2009. Artemisinin resistance in *Plasmodium falciparum* malaria. N Engl J Med 361:455–467. doi:10.1056/NEJMoa0808859. 19641202 PMC3495232

[9] Straimer J, Gnädig NF, Witkowski B, Amaratunga C, Duru V, Ramadani AP, Dacheux M, Khim N, Zhang L, Lam S, Gregory PD, Urnov FD, Mercereau-Puijalon O, Benoit-Vical F, Fairhurst RM, Ménard D, Fidock DA. 2015. K13-propeller mutations confer artemisinin resistance in *Plasmodium falciparum* clinical isolates. Science 347:428–431. doi:10.1126/science.1260867. 25502314 PMC4349400

[10] Ariey F, Witkowski B, Amaratunga C, Beghain J, Langlois A-C, Khim N, Kim S, Duru V, Bouchier C, Ma L, Lim P, Leang R, Duong S, Sreng S, Suon S, Chuor CM, Bout DM, Ménard S, Rogers WO, Genton B, Fandeur T, Miotto O, Ringwald P, Le Bras J, Berry A, Barale J-C, Fairhurst RM, Benoit-Vical F, Mercereau-Puijalon O, Ménard D. 2014. A molecular marker of artemisinin-resistant *Plasmodium falciparum* malaria. Nature 505:50–55. doi:10.1038/nature12876. 24352242 PMC5007947

[11] Gupta VA, Beggs AH. 2014. Kelch proteins: emerging roles in skeletal muscle development and diseases. Skelet Muscle 4:1112. doi:10.1186/2044-5040-4-11. PMC4067060 24959344

[12] Haldar K, Bhattacharjee S, Safeukui I. 2018. Drug resistance in *Plasmodium*. Nat Rev Microbiol 16:156–170. doi:10.1038/nrmicro.2017.161. 29355852 PMC6371404

[13] Siddiqui FA, Boonhok R, Cabrera M, Mbenda HGN, Wang M, Min H, Liang X, Qin J, Zhu X, Miao J, Cao Y, Cui L. 2020. Role of *Plasmodium falciparum* Kelch 13 protein mutations in *P. falciparum* populations from northeastern Myanmar in mediating artemisinin resistance. mBio 11:e01134-19. doi:10.1128/mBio.01134-19. 32098812 PMC7042691

[14] Anderson TJC, Nair S, McDew-White M, Cheeseman IH, Nkhoma S, Bilgic F, McGready R, Ashley E, Pyae Phyo A, White NJ, Nosten F. 2017. Population parameters underlying an ongoing soft sweep in Southeast Asian malaria parasites. Mol Biol Evol 34:131–144. doi:10.1093/molbev/msw228. 28025270 PMC5216669

[15] Imwong M, Suwannasin K, Kunasol C, Sutawong K, Mayxay M, Rekol H, Smithuis FM, Hlaing TM, Tun KM, van der Pluijm RW, Tripura R, Miotto O, Menard D, Dhorda M, Day NPJ, White NJ, Dondorp AM. 2017. The spread of artemisinin-resistant *Plasmodium falciparum* in the Greater Mekong subregion: a molecular epidemiology observational study. Lancet Infect Dis 17:491–497. doi:10.1016/S1473-3099(17)30048-8. 28161569 PMC5406483

[16] Jacob CG, Thuy-Nhien N, Mayxay M, Maude RJ, Quang HH, Hongvanthong B, Vanisaveth V, Duc TN, Rekol H, van der Pluijm RW. 2021. Genetic surveillance in the Greater Mekong Subregion and South Asia to support malaria control and elimination. medRxiv doi:10.1101/2020.07.23.20159624. PMC8354633 34372970

[17] Demas AR, Sharma AI, Wong W, Early AM, Redmond S, Bopp S, Neafsey DE, Volkman SK, Hartl DL, Wirth DF. 2018. Mutations in *Plasmodium falciparum* actin-binding protein coronin confer reduced artemisinin susceptibility. Proc Natl Acad Sci USA 115:12799–12804. doi:10.1073/pnas.1812317115. 30420498 PMC6294886

[18] Wang Z, Cabrera M, Yang J, Yuan L, Gupta B, Liang X, Kemirembe K, Shrestha S, Brashear A, Li X, Porcella SF, Miao J, Yang Z, Su X-Z, Cui L. 2016. Genome-wide association analysis identifies genetic loci associated with resistance to multiple antimalarials in *Plasmodium falciparum* from China-Myanmar border. Sci Rep 6:3389112. doi:10.1038/srep33891. PMC5046179 27694982

[19] Henrici RC, van Schalkwyk DA, Sutherland CJ. 2019. Modification of *pfap2μ* and *pfubp1* markedly reduces ring-stage susceptibility of *Plasmodium falciparum* to artemisinin *in vitro*. Antimicrob Agents Chemother 64:e01542-19. doi:10.1128/AAC.01542-19. 31636063 PMC7187599

[20] Miotto O, Amato R, Ashley EA, MacInnis B, Almagro-Garcia J, Amaratunga C, Lim P, Mead D, Oyola SO, Dhorda M, Imwong M, Woodrow C, Manske M, Stalker J, Drury E, Campino S, Amenga-Etego L, Thanh T-NN, Tran HT, Ringwald P, Bethell D, Nosten F, Phyo AP, Pukrittayakamee S, Chotivanich K, Chuor CM, Nguon C, Suon S, Sreng S, Newton PN, Mayxay M, Khanthavong M, Hongvanthong B, Htut Y, Han KT, Kyaw MP, Faiz MA, Fanello CI, Onyamboko M, Mokuolu OA, Jacob CG, Takala-Harrison S, Plowe CV, Day NP, Dondorp AM, Spencer CCA, McVean G, Fairhurst RM, White NJ, Kwiatkowski DP. 2015. Genetic architecture of artemisinin-resistant *Plasmodium falciparum*. Nat Genet 47:226–234. doi:10.1038/ng.3189. 25599401 PMC4545236

[21] Witkowski B, Amaratunga C, Khim N, Sreng S, Chim P, Kim S, Lim P, Mao S, Sopha C, Sam B, Anderson JM, Duong S, Chuor CM, Taylor WRJ, Suon S, Mercereau-Puijalon O, Fairhurst RM, Menard D. 2013. Novel phenotypic assays for the detection of artemisinin-resistant *Plasmodium falciparum* malaria in Cambodia: *in-vitro* and *ex-vivo* drug-response studies. Lancet Infect Dis 13:1043–1049. doi:10.1016/S1473-3099(13)70252-4. 24035558 PMC5015432

[22] Teuscher F, Gatton ML, Chen N, Peters J, Kyle DE, Cheng Q. 2010. Artemisinin-induced dormancy in *Plasmodium falciparum*: duration, recovery rates, and implications in treatment failure. J Infect Dis 202:1362–1368. doi:10.1086/656476. 20863228 PMC2949454

[23] Meshnick SR, Yang YZ, Lima V, Kuypers F, Kamchonwongpaisan S, Yuthavong Y. 1993. Iron-dependent free radical generation from the antimalarial agent artemisinin (qinghaosu). Antimicrob Agents Chemother 37:1108–1114. doi:10.1128/AAC.37.5.1108. 8517699 PMC187911

[24] Gopalakrishnan AM, Kumar N. 2015. Antimalarial action of artesunate involves DNA damage mediated by reactive oxygen species. Antimicrob Agents Chemother 59:317–325. doi:10.1128/AAC.03663-14. 25348537 PMC4291367

[25] Wang J, Zhang C-J, Chia WN, Loh CCY, Li Z, Lee YM, He Y, Yuan L-X, Lim TK, Liu M, Liew CX, Lee YQ, Zhang J, Lu N, Lim CT, Hua Z-C, Liu B, Shen H-M, Tan KSW, Lin Q. 2015. Haem-activated promiscuous targeting of artemisinin in *Plasmodium falciparum*. Nat Commun 6:1011111. doi:10.1038/ncomms10111. PMC4703832 26694030

[26] Suresh N, Haldar K. 2018. Mechanisms of artemisinin resistance in *Plasmodium falciparum* malaria. Curr Opin Pharmacol 42:46–54. doi:10.1016/j.coph.2018.06.003. 30077118 PMC6314025

[27] Bhattacharjee S, Coppens I, Mbengue A, Suresh N, Ghorbal M, Slouka Z, Safeukui I, Tang H-Y, Speicher DW, Stahelin RV, Mohandas N, Haldar K. 2018. Remodeling of the malaria parasite and host human red cell by vesicle amplification that induces artemisinin resistance. Blood 131:1234–1247. doi:10.1182/blood-2017-11-814665. 29363540 PMC5855022

[28] Mok S, Ashley EA, Ferreira PE, Zhu L, Lin Z, Yeo T, Chotivanich K, Imwong M, Pukrittayakamee S, Dhorda M, Nguon C, Lim P, Amaratunga C, Suon S, Hien TT, Htut Y, Faiz MA, Onyamboko MA, Mayxay M, Newton PN, Tripura R, Woodrow CJ, Miotto O, Kwiatkowski DP, Nosten F, Day NPJ, Preiser PR, White NJ, Dondorp AM, Fairhurst RM, Bozdech Z. 2015. Population transcriptomics of human malaria parasites reveals the mechanism of artemisinin resistance. Science 347:431–435. doi:10.1126/science.1260403. 25502316 PMC5642863

[29] Rawat M, Kanyal A, Sahasrabudhe A, Vembar SS, Lopez-Rubio J-J, Karmodiya K. 2021. Histone acetyltransferase PfGCN5 regulates stress responsive and artemisinin resistance related genes in *Plasmodium falciparum*. Sci Rep 11:1–13. doi:10.1038/s41598-020-79539-w. 33441725 PMC7806804

[30] Mbengue A, Bhattacharjee S, Pandharkar T, Liu H, Estiu G, Stahelin RV, Rizk SS, Njimoh DL, Ryan Y, Chotivanich K, Nguon C, Ghorbal M, Lopez-Rubio J-J, Pfrender M, Emrich S, Mohandas N, Dondorp AM, Wiest O, Haldar K. 2015. A molecular mechanism of artemisinin resistance in *Plasmodium falciparum* malaria. Nature 520:683–687. doi:10.1038/nature14412. 25874676 PMC4417027

[31] Bertolotti A, Zhang Y, Hendershot LM, Harding HP, Ron D. 2000. Dynamic interaction of BiP and ER stress transducers in the unfolded-protein response. Nat Cell Biol 2:326–332. doi:10.1038/35014014. 10854322

[32] Harding HP, Zhang Y, Ron D. 1999. Protein translation and folding are coupled by an endoplasmic-reticulum-resident kinase. Nature 397:271–274. doi:10.1038/16729. 9930704

[33] Zhang M, Gallego-Delgado J, Fernandez-Arias C, Waters NC, Rodriguez A, Tsuji M, Wek RC, Nussenzweig V, Sullivan WJ, Jr. 2017. Inhibiting the *Plasmodium* eIF2α kinase PK4 prevents artemisinin-induced latency. Cell Host Microbe 22:766–776. doi:10.1016/j.chom.2017.11.005. 29241041 PMC5869688

[34] Birnbaum J, Scharf S, Schmidt S, Jonscher E, Hoeijmakers WAM, Flemming S, Toenhake CG, Schmitt M, Sabitzki R, Bergmann B, Fröhlke U, Mesén-Ramírez P, Blancke Soares A, Herrmann H, Bártfai R, Spielmann T. 2020. A Kelch13-defined endocytosis pathway mediates artemisinin resistance in malaria parasites. Science 367:51–59. doi:10.1126/science.aax4735. 31896710

[35] Mizushima N. 2007. Autophagy: process and function. Genes Dev 21:2861–2873. doi:10.1101/gad.1599207. 18006683

[36] Hain AUP, Bosch J. 2013. Autophagy in *Plasmodium*, a multifunctional pathway? Comput Struct Biotechnol J 8:e201308002. doi:10.5936/csbj.201308002. 24688742 PMC3962217

[37] Bansal P, Tripathi A, Thakur V, Mohmmed A, Sharma P. 2017. Autophagy-related protein ATG18 regulates apicoplast biogenesis in apicomplexan parasites. mBio 8:e01468-17. doi:10.1128/mBio.01468-17. 29089429 PMC5666157

[38] Agrawal P, Manjithaya R, Surolia N. 2020. Autophagy‐related protein PfATG18 participates in food vacuole dynamics and autophagy‐like pathway in *Plasmodium falciparum*. Mol Microbiol 113:766–782. doi:10.1111/mmi.14441. 31863491

[39] Pang Y, Yamamoto H, Sakamoto H, Oku M, Mutungi JK, Sahani MH, Kurikawa Y, Kita K, Noda NN, Sakai Y, Jia H, Mizushima N. 2019. Evolution from covalent conjugation to non-covalent interaction in the ubiquitin-like ATG12 system. Nat Struct Mol Biol 26:289–296. doi:10.1038/s41594-019-0204-3. 30911187

[40] Joy S, Thirunavukkarasu L, Agrawal P, Singh A, Sagar BKC, Manjithaya R, Surolia N. 2018. Basal and starvation-induced autophagy mediates parasite survival during intraerythrocytic stages of *Plasmodium falciparum*. Cell Death Discov 4:43. doi:10.1038/s41420-018-0107-9. PMC6170468 30302277

[41] Tomlins AM, Ben-Rached F, Williams RA, Proto WR, Coppens I, Ruch U, Gilberger TW, Coombs GH, Mottram JC, Müller S, Langsley G. 2013. *Plasmodium falciparum* ATG8 implicated in both autophagy and apicoplast formation. Autophagy 9:1540–1552. doi:10.4161/auto.25832. 24025672

[42] Dove SK, Piper RC, McEwen RK, Yu JW, King MC, Hughes DC, Thuring J, Holmes AB, Cooke FT, Michell RH, Parker PJ, Lemmon MA. 2004. Svp1p defines a family of phosphatidylinositol 3,5‐bisphosphate effectors. EMBO J 23:1922–1933. doi:10.1038/sj.emboj.7600203. 15103325 PMC404323

[43] Rieter E, Vinke F, Bakula D, Cebollero E, Ungermann C, Proikas-Cezanne T, Reggiori F. 2013. Atg18 function in autophagy is regulated by specific sites within its β-propeller. J Cell Sci 126:593–604. doi:10.1242/jcs.115725. 23230146

[44] Breglio KF, Amato R, Eastman R, Lim P, Sa JM, Guha R, Ganesan S, Dorward DW, Klumpp-Thomas C, McKnight C, Fairhurst RM, Roberts D, Thomas C, Simon AK. 2018. A single nucleotide polymorphism in the *Plasmodium falciparum atg18* gene associates with artemisinin resistance and confers enhanced parasite survival under nutrient deprivation. Malar J 17:1–16. doi:10.1186/s12936-018-2532-x. 30367653 PMC6204056

[45] Wu H, Ng BSH, Thibault G. 2014. Endoplasmic reticulum stress response in yeast and humans. Biosci Rep 34:e00118. doi:10.1042/BSR20140058. 24909749 PMC4076835

[46] Bridgford JL, Xie SC, Cobbold SA, Pasaje CFA, Herrmann S, Yang T, Gillett DL, Dick LR, Ralph SA, Dogovski C, Spillman NJ, Tilley L. 2018. Artemisinin kills malaria parasites by damaging proteins and inhibiting the proteasome. Nat Commun 9:1–9. doi:10.1038/s41467-018-06221-1. 30228310 PMC6143634

[47] Bernales S, McDonald KL, Walter P. 2006. Autophagy counterbalances endoplasmic reticulum expansion during the unfolded protein response. PLoS Biol 4:e423. doi:10.1371/journal.pbio.0040423. 17132049 PMC1661684

[48] Calfon M, Zeng H, Urano F, Till JH, Hubbard SR, Harding HP, Clark SG, Ron D. 2002. IRE1 couples endoplasmic reticulum load to secretory capacity by processing the XBP-1 mRNA. Nature 415:92–96. doi:10.1038/415092a. 11780124

[49] Yorimitsu T, Nair U, Yang Z, Klionsky DJ. 2006. Endoplasmic reticulum stress triggers autophagy. J Biol Chem 281:30299–30304. doi:10.1074/jbc.M607007200. 16901900 PMC1828866

[50] Ogata M, Hino S-i, Saito A, Morikawa K, Kondo S, Kanemoto S, Murakami T, Taniguchi M, Tanii I, Yoshinaga K, Shiosaka S, Hammarback JA, Urano F, Imaizumi K. 2006. Autophagy is activated for cell survival after endoplasmic reticulum stress. Mol Cell Biol 26:9220–9231. doi:10.1128/MCB.01453-06. 17030611 PMC1698520

[51] Mamidi AS, Ray A, Surolia N. 2019. Structural analysis of *Pf*Sec62-autophagy interacting motifs (AIM) and *Pf*Atg8 interactions for its implications in recovER-phagy in *Plasmodium falciparum*. Front Bioeng Biotechnol 7:240. doi:10.3389/fbioe.2019.00240. 31608276 PMC6773812

[52] Axten JM, Medina JR, Feng Y, Shu A, Romeril SP, Grant SW, Li WHH, Heerding DA, Minthorn E, Mencken T, Atkins C, Liu Q, Rabindran S, Kumar R, Hong X, Goetz A, Stanley T, Taylor JD, Sigethy SD, Tomberlin GH, Hassell AM, Kahler KM, Shewchuk LM, Gampe RT. 2012. Discovery of 7-methyl-5-(1-{[3-(trifluoromethyl) phenyl] acetyl}-2, 3-dihydro-1 H-indol-5-yl)-7 H-pyrrolo [2, 3-d] pyrimidin-4-amine (GSK2606414), a potent and selective first-in-class inhibitor of protein kinase R (PKR)-like endoplasmic reticulum kinase. J Med Chem 55:7193–7207. doi:10.1021/jm300713s. 22827572

[53] Kabeya Y, Mizushima N, Ueno T, Yamamoto A, Kirisako T, Noda T, Kominami E, Ohsumi Y, Yoshimori T. 2000. LC3, a mammalian homologue of yeast Apg8p, is localized in autophagosome membranes after processing. EMBO J 19:5720–5728. doi:10.1093/emboj/19.21.5720. 11060023 PMC305793

[54] Tsuyuki S, Takabayashi M, Kawazu M, Kudo K, Watanabe A, Nagata Y, Kusama Y, Yoshida K. 2014. Detection of WIPI1 mRNA as an indicator of autophagosome formation. Autophagy 10:497–513. doi:10.4161/auto.27419. 24384561 PMC4077887

[55] Proikas-Cezanne T, Ruckerbauer S, Stierhof Y-D, Berg C, Nordheim A. 2007. Human WIPI-1 puncta-formation: a novel assay to assess mammalian autophagy. FEBS Lett 581:3396–3404. doi:10.1016/j.febslet.2007.06.040. 17618624

[56] Dall’Armi C, Devereaux KA, Di Paolo G. 2013. The role of lipids in the control of autophagy. Curr Biol 23:R33–R45. doi:10.1016/j.cub.2012.10.041. 23305670 PMC3587843

[57] Nascimbeni AC, Codogno P, Morel E. 2017. Phosphatidylinositol‐3‐phosphate in the regulation of autophagy membrane dynamics. FEBS J 284:1267–1278. doi:10.1111/febs.13987. 27973739

[58] Burman C, Ktistakis NT. 2010. Regulation of autophagy by phosphatidylinositol 3-phosphate. FEBS Lett 584:1302–1312. doi:10.1016/j.febslet.2010.01.011. 20074568

[59] Petherick KJ, Conway OJL, Mpamhanga C, Osborne SA, Kamal A, Saxty B, Ganley IG. 2015. Pharmacological inhibition of ULK1 kinase blocks mammalian target of rapamycin (mTOR)-dependent autophagy. J Biol Chem 290:11376–11383. doi:10.1074/jbc.C114.627778. 25833948 PMC4416842

[60] Spielmann T, Gras S, Sabitzki R, Meissner M. 2020. Endocytosis in *Plasmodium* and *Toxoplasma* parasites. Trends Parasitol 36:520–532. doi:10.1016/j.pt.2020.03.010. 32340866

[61] Gnädig NF, Stokes BH, Edwards RL, Kalantarov GF, Heimsch KC, Kuderjavy M, Crane A, Lee MCS, Straimer J, Becker K, Trakht IN, Odom John AR, Mok S, Fidock DA. 2020. Insights into the intracellular localization, protein associations and artemisinin resistance properties of *Plasmodium falciparum* K13. PLoS Pathog 16:e1008482. doi:10.1371/journal.ppat.1008482. 32310999 PMC7192513

[62] Yang T, Yeoh LM, Tutor MV, Dixon MW, McMillan PJ, Xie SC, Bridgford JL, Gillett DL, Duffy MF, Ralph SA, McConville MJ, Tilley L, Cobbold SA. 2019. Decreased K13 abundance reduces hemoglobin catabolism and proteotoxic stress, underpinning artemisinin resistance. Cell Rep 29:2917–2928. doi:10.1016/j.celrep.2019.10.095. 31775055

[63] Bakar NA, Klonis N, Hanssen E, Chan C, Tilley L. 2010. Digestive-vacuole genesis and endocytic processes in the early intraerythrocytic stages of *Plasmodium falciparum*. J Cell Sci 123:441–450. doi:10.1242/jcs.061499. 20067995

[64] Slomianny C. 1990. Three-dimensional reconstruction of the feeding process of the malaria parasite. Blood Cells 16:369–378. 2096983

[65] Elliott DA, McIntosh MT, Hosgood HD, Chen S, Zhang G, Baevova P, Joiner KA. 2008. Four distinct pathways of hemoglobin uptake in the malaria parasite *Plasmodium falciparum*. Proc Natl Acad Sci USA 105:2463–2468. doi:10.1073/pnas.0711067105. 18263733 PMC2268159

[66] Dasaradhi PVN, Korde R, Thompson JK, Tanwar C, Nag TC, Chauhan VS, Cowman AF, Mohmmed A, Malhotra P. 2007. Food vacuole targeting and trafficking of falcipain-2, an important cysteine protease of human malaria parasite *Plasmodium falciparum*. Mol Biochem Parasitol 156:12–23. doi:10.1016/j.molbiopara.2007.06.008. 17698213

[67] Ho C-M, Beck JR, Lai M, Cui Y, Goldberg DE, Egea PF, Zhou ZH. 2018. Malaria parasite translocon structure and mechanism of effector export. Nature 561:70–75. doi:10.1038/s41586-018-0469-4. 30150771 PMC6555636

[68] Milani KJ, Schneider TG, Taraschi TF. 2015. Defining the morphology and mechanism of the hemoglobin transport pathway in *Plasmodium falciparum*-infected erythrocytes. Eukaryot Cell 14:415–426. doi:10.1128/EC.00267-14. 25724884 PMC4385801

[69] Tamura N, Oku M, Ito M, Noda NN, Inagaki F, Sakai Y. 2013. Atg18 phosphoregulation controls organellar dynamics by modulating its phosphoinositide-binding activity. J Cell Biol 202:685–698. doi:10.1083/jcb.201302067. 23940117 PMC3747300

[70] Hott A, Casandra D, Sparks KN, Morton LC, Castanares G-G, Rutter A, Kyle DE. 2015. Artemisinin-resistant *Plasmodium falciparum* parasites exhibit altered patterns of development in infected erythrocytes. Antimicrob Agents Chemother 59:3156–3167. doi:10.1128/AAC.00197-15. 25779582 PMC4432152

[71] Coulson RMR, Hall N, Ouzounis CA. 2004. Comparative genomics of transcriptional control in the human malaria parasite *Plasmodium falciparum*. Genome Res 14:1548–1554. doi:10.1101/gr.2218604. 15256513 PMC509263

[72] Nagaraj VA, Sundaram B, Varadarajan NM, Subramani PA, Kalappa DM, Ghosh SK, Padmanaban G. 2013. Malaria parasite-synthesized heme is essential in the mosquito and liver stages and complements host heme in the blood stages of infection. PLoS Pathog 9:e1003522. doi:10.1371/journal.ppat.1003522. 23935500 PMC3731253

[73] Filomeni G, De Zio D, Cecconi F. 2015. Oxidative stress and autophagy: the clash between damage and metabolic needs. Cell Death Differ 22:377–388. doi:10.1038/cdd.2014.150. 25257172 PMC4326572

[74] Stokes BH, Dhingra SK, Rubiano K, Mok S, Straimer J, Gnädig NF, Deni I, Schindler KA, Bath JR, Ward KE, Striepen J, Yeo T, Ross LS, Legrand E, Ariey F, Cunningham CH, Souleymane IM, Gansané A, Nzoumbou-Boko R, Ndayikunda C, Kabanywanyi AM, Uwimana A, Smith SJ, Kolley O, Ndounga M, Warsame M, Leang R, Nosten F, Anderson TJ, Rosenthal PJ, Ménard D, Fidock DA. 2021. *Plasmodium falciparum* K13 mutations in Africa and Asia impact artemisinin resistance and parasite fitness. Elife 10:e66277. doi:10.7554/eLife.66277. 34279219 PMC8321553

[75] Cui L, Wang Z, Miao J, Miao M, Chandra R, Jiang H, Su X, Cui L. 2012. Mechanisms of *in vitro* resistance to dihydroartemisinin in *Plasmodium falciparum*. Mol Microbiol 86:111–128. doi:10.1111/j.1365-2958.2012.08180.x. 22812578 PMC3455110

[76] Trager W, Jensen JB. 1976. Human malaria parasites in continuous culture. Science 193:673–675. doi:10.1126/science.781840. 781840

[77] Lambros C, Vanderberg JP. 1979. Synchronization of *Plasmodium falciparum* erythrocytic stages in culture. J Parasitol 65:418–420. doi:10.2307/3280287. 383936

[78] Crabb BS, Rug M, Gilberger T-W, Thompson JK, Triglia T, Maier AG, Cowman AF. 2004. Transfection of the human malaria parasite *Plasmodium falciparum*, p 263–276. *In* Parasite genomics protocols. Springer, New York, NY. 10.1385/1-59259-793-9:263 15153633

[79] Fidock DA, Wellems TE. 1997. Transformation with human dihydrofolate reductase renders malaria parasites insensitive to WR99210 but does not affect the intrinsic activity of proguanil. Proc Natl Acad Sci USA 94:10931–10936. doi:10.1073/pnas.94.20.10931. 9380737 PMC23535

[80] Tonkin CJ, van Dooren GG, Spurck TP, Struck NS, Good RT, Handman E, Cowman AF, McFadden GI. 2004. Localization of organellar proteins in *Plasmodium falciparum* using a novel set of transfection vectors and a new immunofluorescence fixation method. Mol Biochem Parasitol 137:13–21. doi:10.1016/j.molbiopara.2004.05.009. 15279947

[81] Livak KJ, Schmittgen TD. 2001. Analysis of relative gene expression data using real-time quantitative PCR and the 2^−ΔΔ^*CT* method. Methods 25:402–408. doi:10.1006/meth.2001.1262. 11846609

